# Bile acid metabolomics reveals distinct immunometabolic niches and enables accurate diagnosis of AQP4-IgG–seronegative NMOSD

**DOI:** 10.3389/fimmu.2026.1776159

**Published:** 2026-05-04

**Authors:** Zixin Chen, Yuchen Ye, Yanping Lan, Kengna Fan, Xiaxia Qiu, Renquan Jiang, Xin Yang, Xinyao Yang, Xinrong Lu, Qunfang Huang, Yujue He, Can Liu, Qishui Ou, Zhen Xun

**Affiliations:** 1 Department of Laboratory Medicine, Fujian Key Laboratory of Laboratory Medicine, Gene Diagnosis Research Center, Fujian Clinical Research Center for Clinical Immunology Laboratory Test, The First Affiliated Hospital, Fujian Medical University, Fuzhou, Fujian, China; 2 Department of Laboratory Medicine, National Regional Medical Center, Binhai Campus of the First Affiliated Hospital, Fujian Medical University, Fuzhou, Fujian, China

**Keywords:** AQP4-IgG seronegative, Bas, biomarkers, multiple sclerosis, neuromyelitis optica spectrum disorder

## Abstract

**Background and Objective:**

Differentiating neuromyelitis optica spectrum disorder (NMOSD) from multiple sclerosis (MS) is clinically difficult, especially when patients are seronegative for anti–aquaporin-4 immunoglobulin G (AQP4-IgG). Bile acids (BAs) function as important immunoregulatory metabolites, yet their metabolic signatures have not been profiled across NMOSD subtypes or compared with MS. We therefore profiled BA metabolism in NMOSD and MS to determine diagnostic utility.

**Methods:**

We enrolled 112 NMOSD patients (32 AQP4-IgG seronegative, 80 AQP4-IgG seropositive), 50 MS patients, and 66 healthy controls. Targeted liquid chromatography–mass spectrometry quantified 15 bile acids, from which 71 derived metabolic indices were computed. Candidate biomarkers were identified using nested cross-validation combined with stability selection and integrated into diagnostic models evaluated under a pre-specified internal multi-level validation framework. Associations between BA profiles and neurological disability were assessed using Spearman correlation.

**Results:**

Serum BA signatures differed markedly between NMOSD and MS. NMOSD showed a pronounced increase in primary conjugated BAs, whereas MS displayed enhanced secondary BA metabolism. Notably, AQP4-IgG seronegative NMOSD had significantly lower secondary BA concentrations than all other groups. A BA-based diagnostic model distinguished NMOSD from MS with an AUC of 0.874. When specifically differentiating AQP4-IgG–seronegative NMOSD from MS, deoxycholic acid (DCA) demonstrated strong discriminative potential (AUC 0.965), with internally consistent performance across resampling- and cross-validation–based robustness analyses. Disease-specific correlations between BA profiles and Expanded Disability Status Scale scores were also observed.

**Conclusions:**

Serum bile acid metabolomic profiling reveals disease- and subtype-specific signatures that may assist in the differential diagnosis of NMOSD and MS, particularly in AQP4-IgG–seronegative cases. Given the cross-sectional design, these findings should be interpreted as disease-associated metabolic signatures rather than causal mechanisms. DCA showed strong discriminatory performance; however, its clinical utility remains hypothesis-generating and requires replication in a fully independent external cohort.

## 1 Introduction

Accurately distinguishing neuromyelitis optica spectrum disorder (NMOSD) from multiple sclerosis (MS) is a critical decision point in clinical neurology, with immediate implications for therapy and prognosis (1, 2). This task becomes particularly formidable in patients who are seronegative for anti–aquaporin-4 immunoglobulin G (AQP4-IgG), constituting up to 30% of NMOSD cases (3). In the absence of a definitive biomarker, clinicians must rely on nuanced clinical and radiographic distinctions, often leading to diagnostic delay or error (4, 5), and the attendant risk of treatment escalation with MS therapies that can exacerbate NMOSD. There is therefore an urgent need for objective, biologically grounded tools to augment the diagnostic precision for AQP4-IgG (-) NMOSD.

In this setting, metabolomics offers a new framework to address current diagnostic and mechanistic challenges. Bile acids (BAs), which function as key messengers along the “gut–brain axis”, form a biochemical link between intestinal microbiota and immune regulation within the central nervous system. BAs bidirectionally regulate immunity by activating receptors, such as the farnesoid X receptor (FXR) and G protein-coupled BA receptor 1 (GPBAR1) (6). For example, lithocholic acid (LCA) and its derivatives suppress inflammation by inhibiting NF-κB signaling and shifting macrophage polarization toward an M2 phenotype (7). They also influence adaptive immunity by reducing Th17 cell activity while promoting Treg function, thereby reshaping the overall T-cell (8, 9). Moreover, BAs can cross the blood-brain barrier to reshape neuroimmune microenvironments, such as ursodeoxycholic Acid (UDCA), which blocks neuronal apoptosis and induces microglial polarization toward an anti-inflammatory phenotype (10, 11). In turn, neurological disorders conversely disrupt BA metabolism (12). Stress, inflammation, and autonomic dysfunction caused by neural impairment thus perturb gut microbiota homeostasis, thereby interfering with the co-metabolism of microbial and BAs (13). Disease-related systemic inflammation also impairs liver function and alters expression of key synthesis enzymes, such as CYP7A1, which ultimately reshapes the entire BA pool (14). Given the dynamic dual role of BAs in the gut-brain axis, systematic profiling of their alterations in neurological disorders can offer critical insights for identifying novel diagnostic biomarkers for NMOSD and MS.

Our systematic analysis demonstrates that NMOSD, its clinical subtypes, and MS each exhibit distinct BA profiles. These disease-specific metabolic patterns allow for accurate differentiation between NMOSD and MS, supporting their potential as diagnostic biomarkers independent of AQP4-IgG status. Moreover, several BAs showed strong associations with neurological disability, as reflected by Expanded Disability Status Scale (EDSS) scores. Together, these findings offer a new metabolic perspective and provide candidate biomarkers for improving the precision diagnosis and treatment of NMOSD and MS. Accordingly, we first performed an exploratory analysis to determine whether BA profiles could distinguish NMOSD from MS, and then evaluated serostatus-specific signatures with a particular focus on the diagnostically challenging AQP4-IgG–seronegative subgroup.

## 2 Materials and methods

### 2.1 Patient recruitment

This cross-sectional study was conducted at the First Affiliated Hospital of Fujian Medical University between August 31, 2024, and August 31, 2025, and was approved by the hospital’s Ethics Committee (approval no. MRCTA, ECFAH of FMU [2021]570). We consecutively enrolled hospitalized patients from the Department of Neurology. NMOSD diagnoses followed the 2015 International Consensus Diagnostic Criteria (15), and MS diagnoses were based on the 2018 McDonald Criteria (2). The NMOSD cohort included both AQP4-IgG seropositive and seronegative patients. The inclusion criteria for AQP4-IgG-negative NMOSD required that serum testing (Cell-Based Assay) must rule out both AQP4-IgG and anti-myelin oligodendrocyte glycoprotein IgG. Exclusion criteria included active systemic infection, chronic renal failure, cardiac or hepatic dysfunction, malignancy, and the presence of other neurological or autoimmune disorders beyond NMOSD or MS. To minimize medication-related confounding of BA metabolism, we reviewed electronic prescription records and admission medication reconciliation for the 30 days preceding sampling. Participants were excluded if they had used systemic antibiotics, systemic corticosteroids, proton pump inhibitors (PPIs), statins, or BA–related agents (e.g., UDCA) within 30 days prior to sampling. Healthy controls (HC) were recruited from the outpatient health examination clinic. All HC underwent physical examination and routine laboratory testing, including complete blood count and assessments of hepatic and renal function, to confirm the absence of acute or chronic major physical or psychiatric illness. The HC group was closely matched to both the NMOSD and MS groups in age and sex distribution (P > 0.05).

### 2.2 Sample collection

Peripheral venous blood was obtained from all participants. For NMOSD and MS patients, blood samples were collected during the relapse phase. Relapse was operationally defined as the occurrence of new or worsening neurological symptoms lasting >24 hours, not attributable to infection or fever, and confirmed by an attending neurologist. When available, relapse status was supported by objective evidence, including a ≥1.0-point increase in Expanded Disability Status Scale (EDSS) score and/or new or enlarging T2 lesions or gadolinium-enhancing lesions on MRI. All relapse assessments were independently verified by two attending neurologists. All samples were obtained prior to initiation of acute-phase therapy for the index relapse (e.g., high-dose corticosteroids, intravenous immunoglobulin, or plasma exchange) and no participant had received corticosteroid pulse therapy within 1 month prior to sampling. All participants fasted for ≥ 8 hours before venipuncture. Collected whole blood was allowed to clot at room temperature for 30 minutes and then centrifuged at 1,000–2,000×g for 10 minutes to separate the liquid fraction (serum). The onset-to-sampling interval was <7 days for all included participants. We aliquoted the resulting serum into cryovials and stored them at −80 °C until batch analysis. Trained raters performed neurological assessments on the day of sampling using the Kurtzke EDSS.

### 2.3 Analysis of BAs using liquid chromatography-mass spectrometry

We quantified serum BAs with a validated stable isotope–dilution LC-MS/MS method. In brief, 20 μL of serum underwent protein precipitation with 80 μL of ice-cold methanol containing a panel of deuterated bile-acid internal standards. After centrifugation, we transferred the clarified supernatant to the autosampler for analysis. Chromatographic separation used a reversed-phase C18 column with a gradient elusion; mobile phases consisted of ammonium acetate in water and acetic acid in an organic solvent. Mass spectrometric detection employed electrospray ionization in negative mode with multiple reaction monitoring (MRM).

We defined BA pools and related indices as follows. Primary BAs comprised the sum of taurocholic acid (TCA), taurochenodeoxycholic acid (TCDCA), glycocholic acid (GCA), glycochenodeoxycholic acid (GCDCA), chenodeoxycholic acid (CDCA), and cholic acid (CA). Secondary BAs included deoxycholic acid (DCA), glycodeoxycholic acid (GDCA), taurodeoxycholic acid (TDCA), LCA, glycolithocholic acid (GLCA), taurolithocholic acid (TLCA), UDCA, glycoursodeoxycholic acid (GUDCA), and tauroursodeoxycholic acid (TUDCA). Total bile acids (TBA) represented the combined sum of all quantified primary and secondary BAs (TBA = GDCA + TDCA + GLCA + TLCA + GUDCA + TUDCA + TCA + TCDCA + GCA + GCDCA + CA + CDCA + DCA + LCA + UDCA). We defined conjugated BAs as the sum of TCA, TCDCA, GCA, and GCDCA. Unconjugated BAs consisted of CA, CDCA, DCA, LCA, and UDCA. Glycine-conjugated BAs (G-conjugated BAs) included GCA, GCDCA, GDCA, GLCA, and GUDCA, whereas taurine-conjugated BAs (T-conjugated BAs) comprised TCA, TCDCA, TDCA, TLCA, and TUDCA. We categorized 12-hydroxylated (12-OH) BAs as CA, TCA, GCA, DCA, TDCA, and GDCA. Non-12-OH BAs, included CDCA, TCDCA, GCDCA, LCA, TLCA, GLCA, UDCA, GUDCA, and TUDCA. To characterize BA subgroups, we calculated CA-related BAs (CAs = GCA + TCA + CA), CDCA-related BAs (CDCAs = GCDCA + TCDCA + CDCA), DCA-related BAs (DCAs = GDCA + TDCA + DCA), LCA-related BAs (LCAs = GLCA + TLCA + LCA), and UDCA-related BAs (UDCAs = GUDCA + TUDCA + UDCA). We identified neurotoxic BAs as the sum of GCA, GCDCA, and GDCA, and neuroprotective BAs as all UDCA-related metabolites (UDCAs). The symbol “%” denotes the relative proportion of each index within the overall BA pool.

AQP4-IgG–seronegative NMOSD patients were characterized by a marked relative deficiency of secondary bile acids, defined operationally as a substantial reduction in both absolute concentrations and proportional representation (% of total bile acid pool) of secondary bile acids compared with MS patients and healthy controls, rather than an absolute concentration threshold. For translational operability, we additionally report an ROC-derived decision threshold for DCA (Youden-optimized cutoff) and propose a pragmatic phenotype definition of “secondary BA deficiency” as DCA ≤ 0.068 μmol/L (and/or markedly reduced secondary BA pool indices), pending future standardization against population-based reference ranges.

### 2.4 Machine learning modeling and validation pipeline based on stability selection

To minimize overfitting and to provide transparent estimates of generalizability, we implemented a pre-specified multi-level validation strategy. Specifically, all model development steps (normalization, feature selection, and hyperparameter tuning) were performed exclusively within the training–validation set, while final performance was evaluated on an independent hold-out test set that was not used for any model selection. To develop a robust predictive model, the full dataset was first divided into a training–validation set (n = 129) and an independent hold-out test set (n = 33) using an 8:2 stratified split prior to any feature selection or model fitting, preserving the NMOSD-to-MS ratio. All continuous variables were standardized via Z-score normalization prior to modeling.

Feature Selection via Stability Selection. A nested cross-validation framework with stability selection was employed to identify a robust feature subset while preventing data leakage. An outer 5-fold stratified cross-validation was used for performance estimation. Within each training fold of the outer loop, an inner 5-fold stratified cross-validation was conducted to perform LASSO regression for feature selection. The optimal regularization parameter (λ) was determined using the one-standard-error rule (λ.1se), favoring the most parsimonious model within one standard error of the minimum cross-validation error. The frequency with which a feature was selected across all outer folds defined its stability score. Features with a stability score ≥ 50% were retained to form the final predictor subset.

Model Training and Comparison. Using the selected feature subset, four classifiers—Logistic Regression, Random Forest, Support Vector Machine (SVM) with a linear kernel, and XGBoost—were trained and optimized (via grid search within the inner CV loops). For reference, an additional Logistic Regression model was built using features from a single LASSO run on the entire training–validation set (LASSO+Logistic).

Model performance was assessed through a three-level validation strategy: (1) Stability: Evaluated via 10 repetitions of 5-fold cross-validation on the training–validation set. (2) Robustness: Quantified by deriving 95% confidence intervals for performance metrics from 1,000 bootstrap resamples of the independent test set. (3) Generalizability: Finally assessed on the independent test set to provide an unbiased performance estimate.

A global comparison of model performance based on area under the curve (AUC) was conducted using the Friedman test. When a significant difference was detected, pairwise *post-hoc* comparisons were performed using paired t-tests on 1,000 bootstrap samples, with P-values adjusted via the Holm–Bonferroni correction.

The net clinical benefit of each model across a range of decision thresholds was visualized using decision curve analysis. Quantitative reclassification improvement was measured against the baseline (Logistic Regression with stable features) using the Net Reclassification Index (NRI)and Integrated Discrimination Improvement (IDI).

### 2.5 Statistical analysis

Continuous variables are presented as mean ± standard deviation or median (interquartile range) based on data distribution assessed by the Shapiro–Wilk test; categorical variables are presented as number (percentage). Group comparisons were performed using independent-samples t−test, one−way ANOVA, Mann–Whitney U test, Kruskal–Wallis H test, or Chi−squared test, as appropriate. To explore the overall variation in bile acid profiles, unsupervised principal component analysis (PCA) was applied. Associations between bile acid measures and clinical disability (EDSS scores) were evaluated using Spearman’s rank correlation. AUC and 95% CI were estimated using the nonparametric DeLong method. Robustness was assessed by stratified bootstrap resampling (2,000 iterations) and a label permutation test (5,000 permutations). Our primary objective was to develop a parsimonious diagnostic model based solely on BA-related metabolites to maximize interpretability and translational applicability. Therefore, non-metabolomic clinical variables (including systolic/diastolic blood pressure) were not included as predictors in the primary classification models. This design choice also aimed to avoid model instability and overfitting given the limited sample size and the relatively large number of candidate clinical covariates. A two−tailed P−value < 0.05 was considered statistically significant. All analyses were conducted using Python 3. 14.

## 3 Results

### 3.1 Demographic and clinical characteristics

This study included 112 patients with NMOSD, 50 patients with MS, and 66 HC. The three cohorts were comparable in age and sex distribution, and their baseline demographic characteristics are summarized in **Table 1**. Routine liver function markers (ALT, AST, ALP, γ-GGT and total bilirubin) did not differ significantly among groups. In contrast, systolic and diastolic blood pressure were higher in the NMOSD and MS groups than in HC (SBP *P* = 0.004; DBP *P* < 0.001). Blood pressure was collected as a routine vital sign and is presented here to provide a comprehensive baseline clinical profile of the cohorts.

**Table 1:** Comparison of baseline clinical characteristics among patients with NMOSD, MS, and HC.

Characteristic	NMOSD (n=112)	MS (n=50)	HC (n=66)	*P*-value
Age (years)	37 ± 10	33 ± 12	37 ± 11	0.110
Female (%)	78 (70%)	36 (72%)	44 (66.67%)	0.822
BMI	23.4 ± 3.3	23.7 ± 3.6	22.9 ± 3.1	0.721
EDSS score	3.55 (3.5, 5)	3 (2.5, 3.5)	–	<0.001
Disease duration (year)	5.15 (1.30–15.58)	5.20 (1.10–13.20)	–	0.432
LESCL (%)	95 (84.82%)	4 (8.00%)	–	<0.001
ON (%)	74 (66.07%)	8 (16.00%)	–	<0.001
APS (%)	26 (23.21%)	0 (0.00%)	–	<0.001
TBIL	13.1 (9.9, 15.1)	13.4 (11.2,15.2)	14 (11.7,16.4)	0.132
ALT (U/L)	25 (19, 33)	27 (23, 32)	26 (22, 33)	0.353
AST (U/L)	22 (16, 31)	22 (20, 26)	23 (19, 27)	0.820
Albumin (g/L)	44.2 (43.6, 45.0)	44.5 (44.7, 45.0)	44.7 (43.7, 45.3)	0.884
γ-GGT (U/L)	30 (20, 43)	31 (24, 36)	30 (26, 34)	0.690
ALP (U/L)	78 (63, 97)	84 (74, 89)	80 (72, 93)	0.137
Dyslipidemia (%)	12 (10.71%)	4 (8.00%)	–	0.412
Systolic pressure (mmHg)	122.92 ± 10.73^#^	125.18 ± 12.55^&^	118.18 ± 12.99	0.004
Diastolic pressure (mmHg)	77.79 ± 10.86^#^	79.00 ± 12.02^&^	70.23 ± 9.65	<0.001
History of diabetes (%)	5 (4.46%)	2 (4.00%)	–	0.330
TC	4.8 (4.1, 5.5)	4.6 (3.8, 5.0)	4.6 (4.2, 5.0)	0.117
TG	1.1 (0.8, 1.4)^#**^	0.9 (0.6, 1.1)	0.9 (0.7, 1.1)	0.002
HDL-C	1.3 (1.2, 1.7)	1.4 (1.3, 1.7)	1.4 (1.2, 1.7)	0.438
LDL-C	3.0 (2.6, 3.9)	2.7 (2.3, 3.3)	3.0 (2.6, 3.4)	0.117
ApoA	1.5 (1.2, 1.7)	1.5 (1.2, 1.7)	1.5 (1.2, 1.6)	0.551
ApoB	0.9 (0.7, 1.1) ^#^	0.8 (0.7, 0.9)	0.9 (0.8, 1.0)	0.017
Medications	93 (83%)	44 (88%)	–	0.419
Inrerferon-β1a	–	6 (12%)	–	
Dimethyl fumarate	–	23 (46%)	–	
Fingolimod	–	13 (26%)	–	
Cladribine	–	2 (4%)	–	
Prednisolone	61 (54%)	–	–	
Mycophenolate	32 (29%)	–	–	

BMI, body mass index; EDSS, Expanded Disability Status Scale; LESCL, longitudinally extensive spinal cord lesion; ON, optic neuritis; APS, area postrema syndrome; TBIL, total bilirubin; ALT, alanine aminotransferase; AST, aspartate aminotransferase; γ-GGT, gamma-glutamyl transferase; ALP, alkaline phosphatase; TC, total cholesterol; TG, triglyceride; HDL-C, high-density lipoprotein cholesterol; LDL-C, low-density lipoprotein cholesterol; ApoA, apolipoprotein A; ApoB, apolipoprotein B. *P*-values (last column) were derived from the Kruskal–Wallis H test for intergroup comparisons. ^#^*P* < 0.05 for NMOSD vs MS. **P < 0.01 for NMOSD vs. HC. ^&^*P* < 0.05 for MS vs HC.

Clear distinctions between NMOSD and MS were observed in clinical phenotype and neuroimaging. NMOSD patients exhibited greater neurological disability, with a higher median EDSS score than MS patients (*P* < 0.001). Longitudinally extensive spinal cord lesions and optic neuritis were more frequent in NMOSD than in MS (both *P* < 0.001), and area postrema syndrome occurred in 33.21% of NMOSD patients but in none of the MS patients. As expected, 71.43% of NMOSD patients were AQP4-IgG positive, whereas all MS patients were negative (**Table 1**).

In addition, several lipid-related traits showed modest between-group differences (e.g., TG and ApoB; **Table 1**). To minimize treatment-related confounding, none of the included patients had received corticosteroid pulse therapy within 1 month prior to blood sampling. In addition, the distribution of background disease-modifying therapy (DMT)/immunosuppressant exposure did not differ significantly between NMOSD and MS (*P*>0.05; **Table 1**). Together, these baseline characteristics support clinically representative cohorts and provide the context for downstream BA metabolomic comparisons.

### 3.2 Exploratory BA signatures for differentiating NMOSD from MS

**Figure 1:**
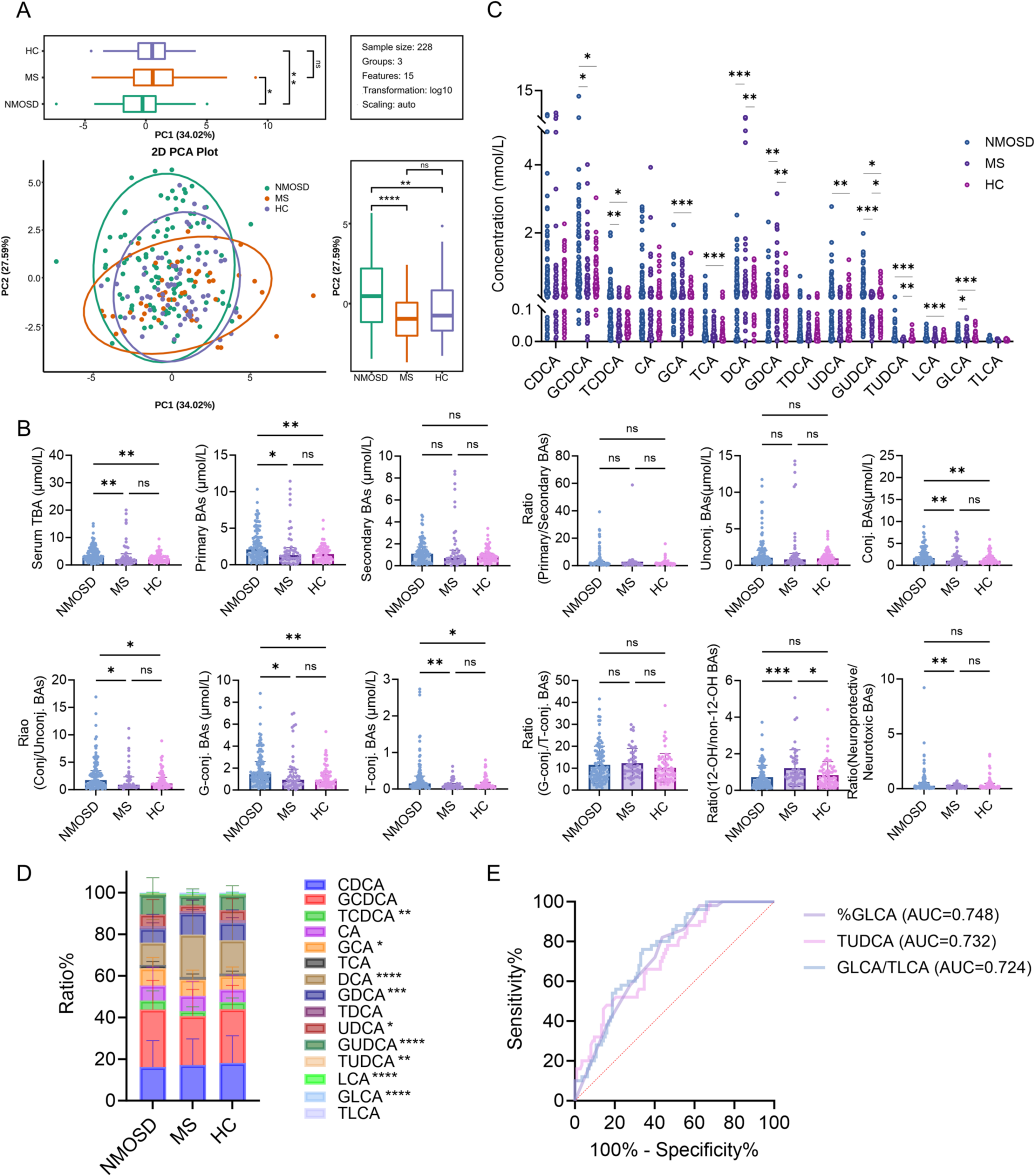
Differential BA metabolomic profiles and their diagnostic value in NMOSD, MS, and HC. **(A)** PCA score plot. **(B)** Overall characteristics of the BA metabolome in NMOSD, MS, and HC. **(C)** Absolute quantification of 15 BA species. **(D)** Relative distribution of BA composition. **(E)** ROC curve of GLCA for discriminating against MS from NMOSD. Data were analyzed using the Kruskal–Wallis H test (**B**-D). *P < 0.05, **P < 0.01, ***P < 0.001, ****P < 0.0001.

Serum BA profiles fundamentally distinguished NMOSD from MS, characterized by a marked shift from primary conjugated BA dominance in NMOSD to secondary BA enrichment in MS. The PCA plot revealed a clear separation along the first principal component between NMOSD patients and the other two groups MS and HC (**Figure 1A**).

Quantitative comparisons uncovered opposing BA metabolic phenotypes (**Figures 1B–D****;** detailed data in **Supplementary Table 1**). NMOSD patients exhibited a significant elevation in TBA concentration, primarily driven by the accumulation of multiple primary conjugated BAs, including glyco- and tauro-conjugates of cholic, chenodeoxycholic, and ursodeoxycholic acids (all *P* < 0.001 vs. MS and HC). Conversely, MS patients displayed a distinct enrichment of secondary BAs, with levels and proportions of DCA, LCA, and their glycine conjugates being significantly higher than those in both NMOSD and HC groups (all *P* < 0.001). Accordingly, the 12-OH/non-12-OH BA ratio, indicative of classical synthesis pathway activity, was significantly higher in MS patients compared to the other groups (*P* < 0.001).

This differential BA signature held direct diagnostic value. Receiver operating characteristic (ROC) curve analysis identified the relative proportion of %GLCA as the most powerful single biomarker for distinguishing MS from NMOSD, yielding an AUC of 0.748 (95% CI: 0.673–0.823, *P* < 0.001; **Figure 1E**, **Supplementary Table 2**).

These results revealed the potential of bile acid profiles for the differential diagnosis of NMOSD and MS.

### 3.3 Machine learning model with stable feature selection for differentiating NMOSD from MS using BA profiling

**Figure 2:**
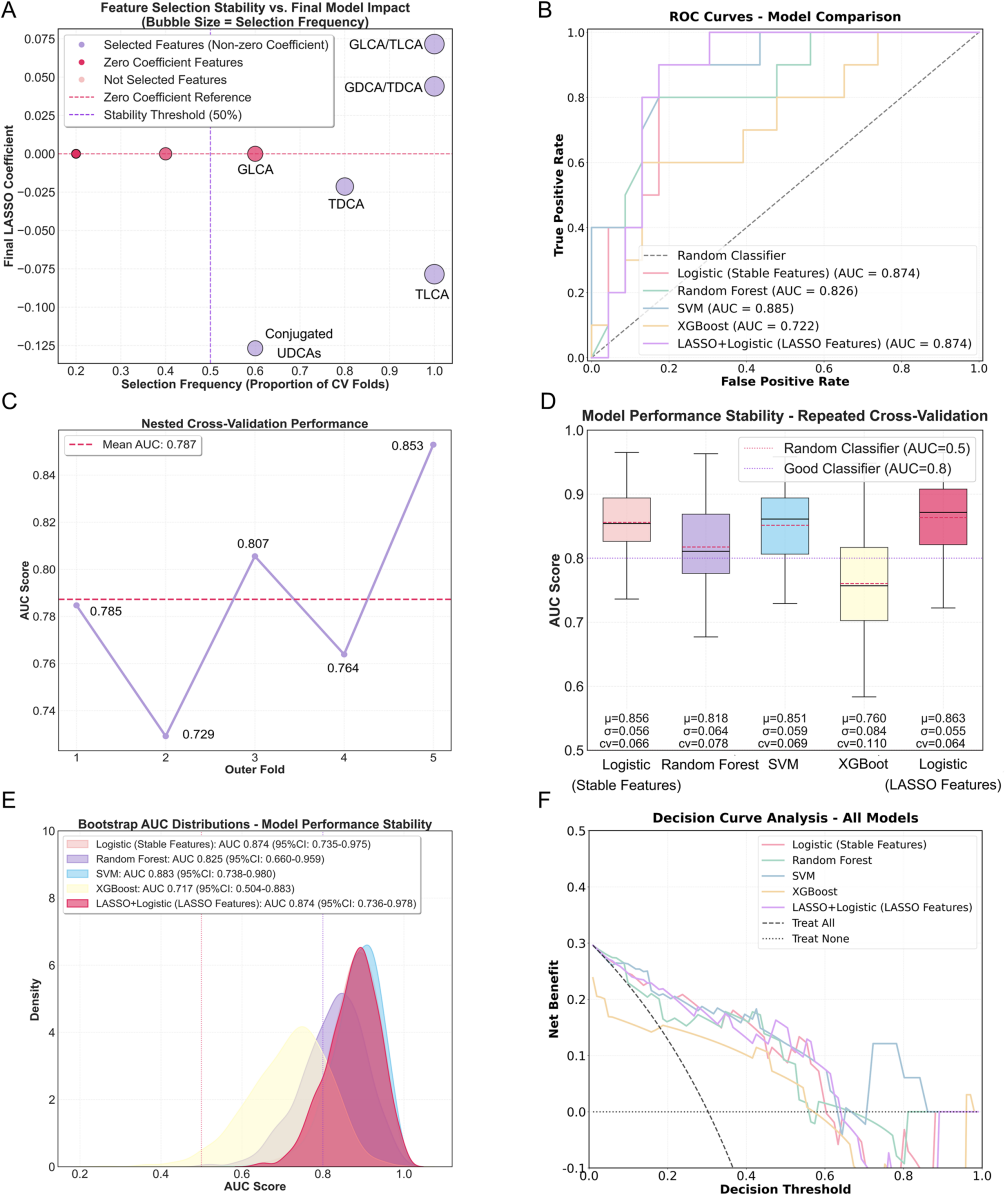
Machine learning model performance and feature stability analysis. **(A)** Feature selection stability versus final model impact. Bubble size represents selection frequency across cross-validation folds. Features in the upper-right quadrant (high stability, high impact) represent the most reliable biomarker candidates in training set. **(B)** ROC curves comparing five machine learning models in test set. **(C)** Nested cross-validation performance of the stability feature-selected Logistic Regression model across five outer folds in training set. **(D)** Repeated cross-validation stability (10 repeats × 5 folds) showing consistent performance across models in training set. **(E)** Bootstrap AUC distributions (1000 resamples) illustrating model performance stability in test set. **(F)** Net benefits of the machine learning models by decision curve analysis for diagnosing NMOSD and MS in test set.

Given the limited diagnostic performance of a single indicator, the study cohort was randomly divided into a training set and a validation set (**Supplementary Table 3**). The LASSO coefficient path and 10-fold cross-validation error plot indicated that the model achieved an optimal balance between predictive accuracy and model complexity (**Supplementary Figures 1A, S1B**). This procedure yielded six stable biomarkers: GLCA, TLCA, TDCA, GLCA/TLCA, GDCA/TDCA and conjugated UDCAs. As shown in **Figure 2A**, GLCA/TLCA, GDCA/TDCA, and TLCA were selected in all outer folds (100% frequency), indicating strong robustness. Examination of the model coefficients showed that GLCA/TLCA (coefficient = 0.072) acted as the strongest positive predictor, whereas conjugated UDCAs (selection frequency = 60%) represented the main negative predictor (coefficient = –0.127).

To identify the optimal model for NMOSD diagnosis, this study also systematically evaluated five machine learning models. First, on the independent test set, the Logistic Regression models (including both Stable Features and LASSO Features) demonstrated excellent and comparable discriminatory performance (AUCs both 0.874) (**Figure 2B**), with high sensitivity (0.900) and good specificity (0.826) (**Table 2**). Although SVM achieved a marginally higher AUC (0.885) and LASSO+Logistic showed better accuracy (0.848) (**Table 2**), statistical significance tests confirmed no significant difference between the two Logistic models (adjusted *P* = 1.000), while both significantly outperformed all other models (adjusted *P* < 0.001) (**Supplementary Table 4**). Confusion matrix analyses (**Supplementary Figure 1C**) further confirmed that classification outcomes were consistent with the performance metrics reported above.

**Table 2:** Comparative Performance Metrics of Machine Learning Models on the Independent Test Set.

Model	AUC	AUC 95%IC		Accuracy	Balanced accuracy	Sensitivity	Specificity	F1_Score	Youden_index	Best_threshold
Logistic (Stable Features)	0.874		0.742-0.974	0.788	0.863	0.9	0.826	0.783	0.726	0.563
Random Forest	0.826		0.662-0.965	0.788	0.835	0.8	0.870	0.762	0.670	0.440
SVM	0.885		0.758-0.978	0.758	0.863	0.9	0.826	0.783	0.726	0.434
XGBoost	0.722		0.508-0.895	0.758	0.735	0.6	0.870	0.632	0.470	0.457
LASSO+Logistic	0.874		0.745-0.980	0.848	0.863	0.9	0.826	0.783	0.726	0.511

All performance metrics are evaluated on the independent test set. Balanced Accuracy, the arithmetic means of sensitivity and specificity, provides a robust metric for class-imbalanced data.

Second, nested cross-validation produced a mean AUC of 0.787 (**Figure 2C**), confirming the model’s reliable generalization ability under a strict validation framework. With regards to model stability, LASSO+Logistic exhibited the best performance in repeated cross-validation (mean AUC = 0.863 ± 0.055, CV = 0.064) (**Figure 2D**, **Supplementary Table 5**), with its stability further validated through Bootstrap resampling (AUC = 0.874, 95% CI: 0.736-0.978) (**Figure 2E**, **Supplementary Table 6**). The model with stable features demonstrated slightly inferior performance comparatively.

However, the ultimate decision-maker for the selected model was due to the clinical utility assessment. When using Logistic Regression (Stable Features) as the baseline for reclassification analysis, all comparative models demonstrated negative NRI values, indicating inferior overall reclassification capability compared to the baseline model (**Supplementary Table 7**). This finding is particularly crucial for ensuring accurate identification of NMOSD patients (event NRI). Decision curve analyses were used to assess the clinical value of the different models (**Figure 2F**). Both the Logistic and SVM models showed favorable net benefit compared to the Random Forest and XGBoost models. Thus, the Logistic Regression (Stable Features) model demonstrated superior clinical reclassification ability while maintaining comparable discriminatory performance, and was selected as the optimal choice for NMOSD diagnosis. Detailed analysis of the Logistic Regression (Stable Features) model is summarized in **Table 3**.

**Table 3:** Logistic Regression Analysis of Stable BA Feature.

Variable	Coefficient (B)	Std. error	Wald statistic	*P*-value	Odds ratio [Exp(B)]	95% CI for exp(B)
Intercept	-0.639	0.260	-2.460	0.014	0.528	0.317-0.878
TLCA	-0.698	0.254	-2.750	0.006	0.498	0.303-0.818
Conjugated UDCAs	-1.120	0.307	-3.650	<0.001	0.326	0.179-0.596
GDCA/TDCA	0.706	0.340	2.076	0.038	2.025	1.040-3.943
GLCA/TLCA	0.744	0.281	2.648	0.008	2.104	1.213-3.650
TDCA	-0.270	0.271	-0.994	0.320	0.764	0.449-1.300
GLCA	-0.218	0.243	-0.895	0.371	0.804	0.499-1.296

Logistic regression model with stable feature selection. Coefficient (B) indicates the log-odds change per unit increase in the standardized variable. Odds Ratio [Exp(B)] represents the multiplicative change in odds per unit increase.

### 3.4 Differentiating MS from AQP4-IgG negative NMOSD using BA profiles

**Figure 3:**
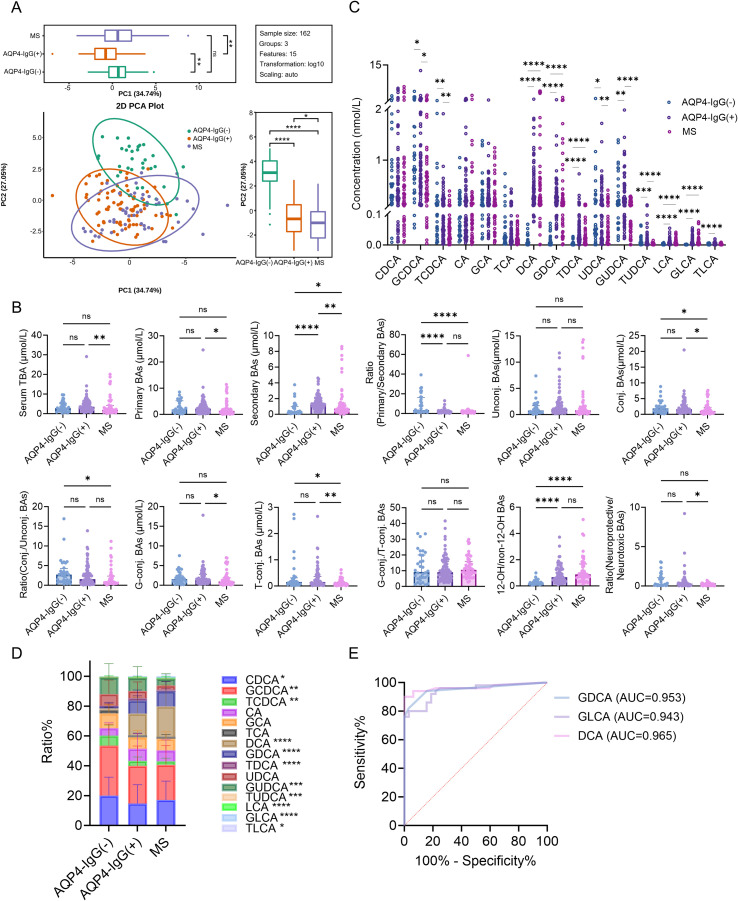
Differential BA metabolomic profiles and their diagnostic value in AQP4-IgG (-), AQP4-IgG (+), and MS. **(A)** PCA score plot. **(B)** Overall characteristics of the BA metabolome. **(C)** Absolute quantification of 15 BA species. **(D)** Relative distribution of BA composition. **(E)** Receiver operating characteristic curve of DCA for discriminating between AQP4-IgG (-) andMS. Data were analyzed using the Kruskal–Wallis H test **(B–D)**. *P < 0.05, **P < 0.01, ***P < 0.001, ****P < 0.0001.

To directly address the diagnostic challenge in seronegative patients, we compared BA metabolism across 50 MS patients, 80 AQP4-IgG seropositive, and 32 AQP4-IgG seronegative NMOSD patients (clinical characteristics in **Supplementary Table 8**). PCA showed separation among the three cohorts, with their positions along the principal components suggesting a potential ordering (**Figure 3A**). Quantitative comparisons revealed distinct BA metabolic phenotypes: (i) pronounced suppression of secondary BAs, (ii) dominance of conjugated BAs with intermediate levels of secondary BAs, and (iii) elevated abundance of secondary BAs.

AQP4-IgG (–) NMOSD patients were characterized by a profound suppression of secondary BAs. Compared with both AQP4-IgG (+) NMOSD and MS groups, the seronegative NMOSD cohort had significantly lower levels of total secondary BAs (all *P* < 0.001), driven by markedly reduced concentrations and relative proportions of DCA, GDCA, LCA, and GLCA (all *P* < 0.001) (**Figures 3B–D****;****Supplementary Table 9**).

AQP4-IgG (+) NMOSD patients occupied an intermediate position, characterized by a clear predominance of conjugated BAs. Their TBA concentration was significantly expanded compared to MS patients (*P* < 0.05), a shift primarily attributable to a pronounced increase in primary conjugated BAs. While secondary BA levels in this group were substantially higher than in their seronegative NMOSD counterparts, they remained consistently lower than those characteristic of the MS cohort, underscoring their intermediate position (**Figure 3B**, **Supplementary Table 9**). Notably, the secondary conjugated BAs GUDCA and TUDCA were significantly enriched in AQP4-IgG (+) NMOSD patients, with concentrations surpassing those in MS patients (all *P* < 0.001) while not differing significantly from the AQP4-IgG (–) NMOSD subgroup (**Figure 3C**, **Supplementary Table 9**). In contrast, MS patients displayed a distinct “secondary BA metabolic advantage”.

Given these pronounced metabolic differences, we assessed the diagnostic utility of these BA metrics in distinguishing MS from AQP4-IgG (–) NMOSD using ROC curve analysis. Several BA parameters showed robust discriminative performance (**Supplementary Table 10**). Among them, DCA emerged as the most powerful single biomarker in the primary ROC analysis (AUC = 0.965; 95% CI: 0.924–1.000; *P* < 0.001; **Figure 3E**). At the Youden index–optimized cutoff (DCA ≤ 0.068 μmol/L), DCA achieved 100% sensitivity and 90% specificity for identifying AQP4-IgG(–) NMOSD (**Table 4**). Because such near-perfect discrimination can be optimistic in modest sample sizes, we further assessed the internal robustness of DCA using resampling- and permutation-based validation procedures.

**Table 4:** ROC-derived optimal cutoff and diagnostic performance of DCA (μmol/L) for differentiating MS from AQP4-IgG(–) NMOSD.

Biomarker	ACU	AUC 95% CI	Accuracy	Balanced accuracy	Sensitivity	Specificity	F1 score	Youden index	Best threshold (μmol/L
DCA	0.965	0.925–1.000	0.939	0.950	1.000	0.900	0.928	0.900	0.068

AUC 95% confidence intervals were calculated using the nonparametric DeLong method. The optimal cutoff was selected by maximizing Youden’s index (J = sensitivity + specificity − 1). Classification rule: DCA ≤ 0.068 μmol/L indicates AQP4-IgG(–) NMOSD. The cutoff is derived within the current cohort and should be considered exploratory pending external validation; bootstrap-based stability estimates for the cutoff and related indices are provided in **Supplementary Table 11**.

DCA showed strong discriminatory ability with an AUC of 0.965 (DeLong 95% CI 0.925–1.000). Bootstrap resampling (2,000 iterations) yielded a stable AUC distribution with a percentile 95% interval of 0.922–0.998. A permutation test (5,000 label permutations) confirmed that such performance is unlikely under the null (empirical one-sided *P* = 0.0002). In repeated stratified holdout evaluation (1,000 repeats, 20/80 split), the AUC showed a 95% range of 0.875–1.000. Importantly, in repeated stratified 5-fold cross-validation (200 repetitions), the mean test-set AUC across folds per repetition remained high and stable, with a percentile 95% interval of 0.954–0.975. The Youden-optimized cutoff was stable under stratified bootstrap resampling (**Supplementary Table 11**), supporting the robustness of the proposed decision threshold.

Collectively, these results provide essential metabolomic evidence that may improve the differential diagnosis of MS versus AQP4-IgG (–) NMOSD and offer new insight into their distinct pathophysiological mechanisms.

### 3.5 Disease-specific links between BAs and disability

**Figure 4:**
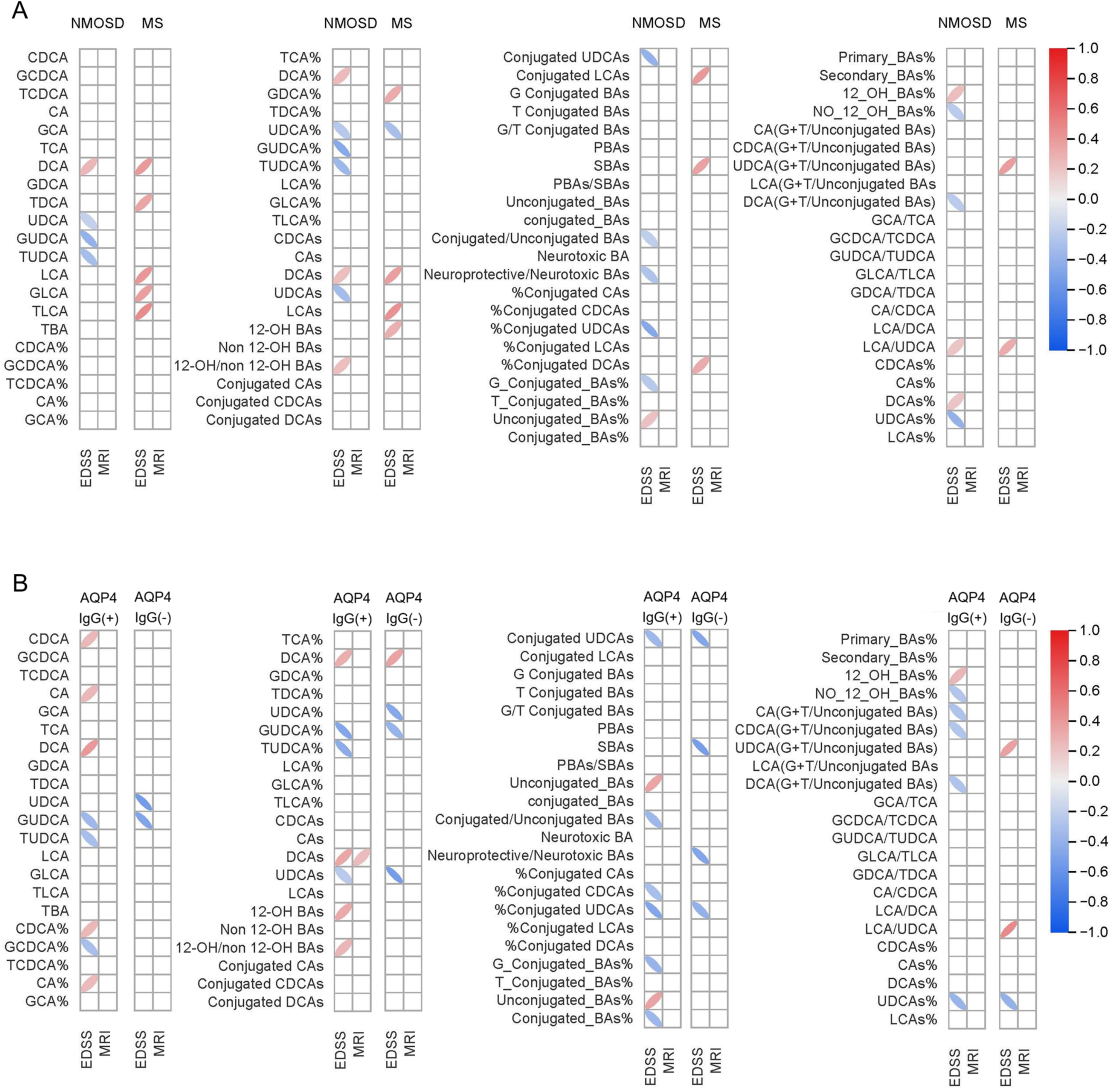
Correlation of BA biomarkers with EDSS scores and spinal cord MRI T2-Lesions in NMOSD and MS. Data were analyzed using Spearman’s rank correlation analysis.

Next, we examined how BAs indicators relate to clinical severity in NMOSD and MS. In NMOSD, several UDCA-related parameters inversely correlated with disability, consistent with a potential neuroprotective or anti-inflammatory effect. In contrast, DCA and its relative proportion showed positive correlations with EDSS scores, suggesting that this putative neurotoxic metabolite may contribute to disease exacerbation. MS patients exhibited a different pattern, with EDSS scores positively correlating with multiple secondary BAs, most notably LCA (**Figure 4A**, **Supplementary Table 12**). Within NMOSD subgroups, correlation patterns with disability were subtly distinct. For example, in the AQP4-IgG-positive subgroup, only DCA showed a weak positive correlation with the number of spinal lesion segments (r = 0.235, *P* = 0.036), whereas no other BA metrics were significantly associated with MRI findings (all *P* > 0.05; **Figure 4B**, **Supplementary Table 13**).

These findings suggest that the two diseases differ in their underlying BA metabolic disturbances and highlight a potential metabolic dimension for differentiating their mechanisms of neural injury.

## 4 Discussion

Our study establishes that serum BA profiles serve as powerful discriminators between NMOSD and MS. Beyond distinguishing the diseases broadly (AUC 0.874), we identify a profound suppression of secondary BAs, particularly DCA, as a hallmark of AQP4-IgG-seronegative NMOSD, enabling near-perfect separation from MS (AUC 0.965). These BA signatures are not merely diagnostic; they correlate in disease-specific ways with neurological disability, implicating distinct immunometabolic pathways in NMOSD and MS pathogenesis.

To reconcile the causality dilemma, we propose a conceptual model of self-reinforcing immunometabolic niches to integrate these observations. The cycle begins with a constitutive host-microbiome signature (influenced by genetics), which establishes a baseline BA profile that pre-configures systemic immunity. Following disease onset, the activated, disease-specific immune response then feedbacks to amplify and lock in the initial metabolic dysregulation (16). Below, we define three distinct niches arising from this dynamic. This framework is intended to be hypothesis-generating rather than causal, and longitudinal studies will be required to determine temporal directionality.

First, AQP4-IgG–seronegative NMOSD resides in a “metabolically quiescent” immunometabolic niche, characterized by reduced microbiota-derived secondary BA signaling, which could predispose to a distinct immunophenotype. Currently, no clinically validated cutoff values exist for defining secondary bile acid deficiency; thus, the term is used descriptively to denote a disease-specific metabolic pattern relevant for future translational investigation. This paucity of immunomodulatory bacterial metabolites may reflect or contribute to diminished host-microbiota crosstalk. Given the established role of such BAs in shaping T-helper cell responses (8, 17), their scarcity could underlie a distinct, less Th17-driven immune pathophysiology that characterizes this NMOSD subtype, potentially explaining its unique clinical and therapeutic profile.

In contrast, AQP4-IgG–seropositive NMOSD occupies a niche of “tense balance” between injury and repair. We observed a compelling metabolic dichotomy: a concurrent rise in neuroprotective UDCAs (e.g., TUDCA, known to mitigate ER stress and apoptosis (18, 19)) and pro-inflammatory primary BAs (e.g., GCDCA (20, 21)). We hypothesize that the humoral immunity and profound astrocytopathy characteristic of this subtype may contribute to this metabolic imbalance. The elevated UDCAs may represent an endogenous neuroprotective response to astrocyte injury—a built-in defense mechanism with clear therapeutic implications. Simultaneously, systemic inflammation likely promotes the observed shift toward conjugated primary BAs, fueling a self-reinforcing cycle of injury and compromised repair.

Finally, MS is characterized by a “pro-inflammatory priming” niche, marked by a pronounced hypermetabolism of secondary BAs. This profile provides direct metabolomic support for the gut–brain axis hypothesis in MS. Elevated levels of microbiota-derived BAs like DCA, which can promote Th17 differentiation (8, 22, 23), may establish a feed-forward loop: MS-related T-cell inflammation might alters gut permeability and microbiota composition, possibly favoring bacteria that produce Th17-promoting metabolites, which could in turn sustain or amplify neuroinflammation.

The initial non-stratified analysis was intended as an exploratory step to establish disease-level BA differences between NMOSD and MS under real-world clinical conditions where serostatus may not be immediately available. These results provided a necessary framework for the subsequent antibody-specific analyses by demonstrating that BA dysregulation is a shared but heterogeneous feature within the NMOSD spectrum. These metabolically defined niches are diagnostically actionable. Our BA-based model outperforms previous metabolic or protein-based approaches, offering a non-invasive strategy particularly valuable in seronegative cases. For instance, previous attempts using serum neurofilament light chain or cerebrospinal fluid metabolites, such as kynurenine pathway products, yielded moderate accuracy (AUCs 0.70–0.82) in differentiating NMOSD from MS (24, 25). In contrast, our BA-based model utilizes stable serum metabolites, offering a non-invasive yet discriminative approach, particularly in the challenging seronegative NMOSD subgroup, where DCA alone achieved an AUC of 0.965. Importantly, the diagnostic performance of DCA in distinguishing AQP4-IgG–seronegative NMOSD from MS should be considered preliminary and requires validation in independent, external cohorts to exclude overfitting and center-specific effects. Nevertheless, the robustness analyses (bootstrap resampling, label permutation, and repeated stratified cross-validation) showed a consistently high and stable AUC distribution, supporting that the observed signal is unlikely to be a chance finding.

The disease-specific correlations between BA profiles and disability scores further support their pathophysiological relevance and potential utility as dynamic biomarkers for neuroinflammatory activity (5, 26). Beyond NMOSD and MS, BA dysregulation has been increasingly linked to other neurological disorders. In Alzheimer’s disease, reduced levels of taurine-conjugated BAs have been associated with cognitive decline (27); and in Parkinson’s disease, altered secondary BA ratios correlate with disease severity and progression (28). These findings suggest that BA profiling may serve as a shared metabolic window into neuroinflammatory and neurodegenerative processes across a spectrum of neuroinflammatory diseases. Nevertheless, metabolic alterations may persist beyond the acute inflammatory window, and subclinical disease activity cannot be fully excluded in a cross-sectional design. Therefore, the observed BA profiles should be interpreted as disease-state–associated metabolic signatures rather than strictly acute-phase inflammatory markers.

While our findings highlight the potential of BA metabolomics, several limitations should be noted. First, the cross-sectional design precludes causal inference between altered BA metabolism and disease mechanisms. Second, the absence of external multi-center validation and the single-center, relatively homogeneous cohort constrain generalizability; thus, model performance should be interpreted as internally validated and hypothesis-generating, as nested cross-validation, bootstrap resampling, and an internal hold-out set cannot substitute for independent multi-center verification before clinical translation. Third, despite screening major medication classes affecting bile acid metabolism and excluding recent exposure within 30 days, residual confounding from longer-term medication histories and heterogeneous background DMT/immunosuppressant regimens may persist and could influence microbiota and BA profiles. Blood pressure was collected for cohort characterization but not included in the primary BA-only models; therefore, confounding by systemic/hemodynamic factors cannot be fully excluded. Future studies should prioritize external multi-center validation and incorporate longitudinal sampling to clarify how BA profiles relate to disease activity, progression, and prognostic outcomes.

In conclusion, we delineate distinct, clinically relevant BA metabolomic signatures that accurately differentiate NMOSD from MS and its serological subtypes. The notable performance of DCA in identifying seronegative NMOSD aids in addressing a pressing diagnostic challenge. By framing these findings within the concept of self-reinforcing immunometabolic niches, we provide a novel pathophysiological perspective and a compelling rationale for developing BA-based diagnostic and monitoring tools in clinical neurology.

## Data Availability

The raw data supporting the conclusions of this article will be made available by the authors, without undue reservation.
