# Supplementary material for: Bile acid metabolomics reveals distinct immunometabolic niches and enables accurate diagnosis of AQP4-IgG–seronegative NMOSD
Source: Front Immunol. 2026 May 4;17:1776159. doi: 10.3389/fimmu.2026.1776159 (PMC13180569; doi:10.3389/fimmu.2026.1776159)
Supplement: Supplementary file 1 [file DataSheet1.docx]

**Bile Acid Metabolomics Reveals Distinct Immunometabolic Niches and Enables Accurate Diagnosis of AQP4-IgG–Seronegative NMOSD**

**Authors:** Zixin Chen^1#^, Yuchen Ye^1,2#^, Yanping Lan^1#^, Kengna Fan^1,2^, Xiaxia Qiu^1^, Renquan Jiang^1,2^, Xin Yang^1^, Xinyao Yang^1^, Xinrong Lu^1^, Qunfang Huang^1,2^, Yujue He^1,2^, Can Liu^1,2^, Qishui Ou^1,2*^, Zhen Xun^1,2*^

1 Department of Laboratory Medicine, Fujian Key Laboratory of Laboratory Medicine, Gene Diagnosis Research Center, Fujian Clinical Research Center for Clinical Immunology Laboratory Test, The First Affiliated Hospital, Fujian Medical University, Fuzhou, Fujian Province, China
2 Department of Laboratory Medicine, National Regional Medical Center, Binhai Campus of the First Affiliated Hospital, Fujian Medical University, Fuzhou, Fujian Province, China

# These authors contributed equally to this work.
*** Corresponding author:** Qishui Ou, MD, PhD, Department of Laboratory Medicine, The First Affiliated Hospital, Fujian Medical University, Fuzhou 350005, Fujian, China. E-mail: ouqishui@fjmu.edu.cn; or Zhen Xun, MD, PhD, Department of Laboratory Medicine, The First Affiliated Hospital, Fujian Medical University, Fuzhou 350005, Fujian, China. E-mail: xunzhen@fjmu.edu.cn. Tel: +86-591-87981909; Fax: 86-591-83340702.

**Supplementary Tables**

**Table S1. Comparison of BA Metabolic Profiles among NMOSD, MS, and Healthy Control Groups**

| **BA** | **NMOSD (n=112)** | **MS (n=50)** | **HC (n=66)** | ***P*-value** |
| --- | --- | --- | --- | --- |
| CDCA (μmol/L) | 0.373(0.197, 0.873) | 0.259(0.084, 0.741) | 0.335(0.132, 0.787) | 0.314 |
| GCDCA (μmol/L) | 0.775(0.434, 1.428) ^*#^ | 0.507(0.232, 0.984) | 0.501(0.320, 0.849) | 0.004 |
| TCDCA (μmol/L) | 0.090(0.373, 0.206) ^**#^ | 0.047(0.019, 0.078) | 0.058(0.033, 0.109) | <0.001 |
| CA (μmol/L) | 0.089(0.433, 0.322) | 0.098(0.036, 0.289) | 0.062(0.029, 0.161) | 0.118 |
| GCA (μmol/L) | 0.218(0.119, 0.413) ^###^ | 0.130(0.074, 0.337) | 0.105(0.063, 0.240) | <0.001 |
| TCA (μmol/L) | 0.255(0.012, 0.060) ^##^ | 0.018(0.010, 0.037) | 0.013(0.006, 0.032) | 0.002 |
| UDCA (μmol/L) | 0.096(0.024, 0.261) ^**^ | 0.150(0.029, 0.160) | 0.072(0.029, 0.160) | 0.003 |
| GUDCA (μmol/L) | 0.185(0.088, 0.494) ^****#^ | 0.077(0.043, 0.174) ^&^ | 0.122(0.065, 0.239) | <0.001 |
| TUDCA (μmol/L) | 0.006(0.002, 0.018) ^***^ | 0.002(0.001, 0.005) ^&&^ | 0.005(0.002, 0.008) | <0.001 |
| DCA (μmol/L) | 0.224(0.036, 0.540) ^***^ | 0.268(0.142, 0.648) ^&&^ | 0.236(0.127, 0.447) | <0.001 |
| GDCA (μmol/L) | 0.134(0.028, 0.294) ^**^ | 0.159(0.072, 0.365) ^&&^ | 0.138(0.055, 0.277) | 0.003 |
| TDCA (μmol/L) | 0.022(0.004, 0.049) | 0.021(0.008, 0.050) | 0.023(0.010, 0.041) | 0.903 |
| LCA (μmol/L) | 0.004(0.002, 0.010) ^###^ | 0.007(0.003, 0.017) | 0.009(0.004, 0.016) | 0.013 |
| GLCA (μmol/L) | 0.003(0.001, 0.010) ^****#^ | 0.010(0.004, 0.021) | 0.008(0.003, 0.013) | <0.001 |
| TLCA (μmol/L) | 0.002(0.001, 0.006) | 0.002(0.001, 0.003) | 0.002(0.001, 0.003) | 0.881 |
| TBA (μmol/L) | 3.217(2.030, 5.409) ^**##^ | 1.961(1.210, 4.083) | 2.195(1.409, 3.160) | 0.001 |
| %CDCA | 0.132(0.058, 0.230) | 0.146(0.060, 0.284) | 0.160(0.069, 0.296) | 0.629 |
| %GCDCA | 0.256(0.184, 0.369) | 0.211(0.121, 0.327) | 0.268(0.174, 0.340) | 0.183 |
| %TCDCA | 0.029(0.014, 0.063) ^**^ | 0.019(0.009, 0.034) ^&&^ | 0.030(0.020, 0.047) | 0.002 |
| %CA | 0.035(0.016, 0.086) | 0.046(0.029, 0.091) | 0.029(0.018, 0.069) | 0.083 |
| %GCA | 0.071(0.049, 0.115) ^#^ | 0.071(0.046, 0.093) | 0.054(0.034, 0.090) | 0.03 |
| %TCA | 0.009(0.004, 0.096) | 0.009(0.005, 0.013) | 0.007(0.003, 0.015) | 0.101 |
| %DCA | 0.073(0.022, 0.157) ^****##^ | 0.171(0.100, 0.295) | 0.129(0.059, 0.242) | <0.001 |
| %GDCA | 0.051(0.013, 0.094) ^***^ | 0.101(0.047, 0.129) | 0.074(0.032, 0.122) | <0.001 |
| %TDCA | 0.007(0.002, 0.017) | 0.011(0.005, 0.017) | 0.012(0.005, 0.021) | 0.061 |
| %UDCA | 0.025(0009, 0.071) * | 0.021(0.007, 0.050) | 0.033(0.014, 0.064) | 0.044 |
| %GUDCA | 0.073(0.030, 0.127) ^****#^ | 0.031(0.017, 0.056) ^&&^ | 0.056(0.034, 0.079) | <0.001 |
| %TUDCA | 0.002(0.001, 0.006) ^***^ | 0.001(0.001, 0.002) ^&&^ | 0.002(0.002, 0.003) | <0.001 |
| %LCA | 0.001(<0.001, 0.004) ^***####^ | 0.003(0.002, 0.006) | 0.004(0.001, 0.008) | <0.001 |
| %GLCA | 0.001(<0.001, 0.004) ^****###^ | 0.006(0.002, 0.009) | 0.003(0.001, 0.008) | <0.001 |
| %TLCA | 0.001(<0.001, 0.002) | 0.001(0.001, 0.002) | 0.001(0.001, 0.002) | 0.062 |
| CDCAs (μmol/L) | 1.466(0.805, 2.793) ^*#^ | 0.925(0.473, 1.653) | 1.117(0.628, 1.676) | 0.004 |
| CAs (μmol/L) | 0.479(0.225, 0.872) ^###^ | 0.318(0.137, 0.856) | 0.258(0.139, 0.422) | 0.001 |
| DCAs (μmol/L) | 0.488(0.088, 0.998) | 0.495(0.263, 1.250) | 0.482(0.210, 0.801) | 0.267 |
| UDCAs (μmol/L) | 0.306(0.1312, 0.847) ^****^ | 0.123(0.069, 0.306) | 0.207(0.112, 0.417) | <0.001 |
| LCAs (μmol/L) | 0.011(0.005, 0.025) ^*#^ | 0.021(0.009, 0.037) | 0.022(0.010, 0.030) | 0.005 |
| 12-OH BAs (μmol/L) | 0.981(0.547, 1.824) | 0.832(0.398, 2.441) | 0.836(0.457, 1.133) | 0.171 |
| Non-12-OH BAs (μmol/L) | 2.056(1.129, 3.704) ^***##^ | 1.159(0.542, 1.992) | 1.377(0.775, 2.171) | <0.001 |
| 12-OH/Non-12-OH BAs | 0.485(0.270, 1.010) ^***^ | 0.872(0.628, 1.675) ^&^ | 0.650(0.343, 1.062) | <0.001 |
| Conjugated CAs (μmol/L) | 0.252(0.129, 0.491) ^###^ | 0.150(0.088, 0.426) | 0.123(0.073, 0.266) | <0.001 |
| Conjugated CDCAs (μmol/L) | 0.859(0.519, 1.739) ^**#^ | 0.565(0.253, 1.075) | 0.574(0.382, 0.922) | 0.002 |
| Conjugated DCAs (μmol/L) | 0.170(0.0348, 0.351) | 0.186(0.083, 0.413) | 0.164(0.065, 0.306) | 0.417 |
| Conjugated UDCAs (μmol/L) | 0.193(0.093, 0.512) ^****#^ | 0.076(0.044, 0.179) | 0.127(0.067, 0.247) | <0.001 |
| Conjugated LCAs (μmol/L) | 0.007(0.003, 0.014) ^*^ | 0.012(0.005, 0.024) | 0.010(0.005, 0.018) | 0.020 |
| G-Conjugated BAs (μmol/L) | 1.460(0.885, 2.652) ^*##^ | 0.926(0.470, 1.898) | 0.853(0.604, 1.581) | 0.001 |
| T-Conjugated BAs (μmol/L) | 0.155(0.077, 0.354) ^**^ | 0.090(0.045, 0.154) | 0.102(0.060, 0.187) | 0.002 |
| G/T-Conjugated BAs | 9.298(5.374, 16.100) | 10.580(7.614, 15.970) | 8.856(5.259, 13.910) | 0.151 |
| Primary BAs (μmol/L) | 2.077(1.236, 3.760) ^*##^ | 1.305(0.694, 2.334) | 1.468(0.775, 2.151) | 0.001 |
| Secondary BAs (μmol/L) | 1.076(0.507, 1.708) | 0.706(0.411, 1.424) | 0.838(0.519, 1.147) | 0.161 |
| Primary/Secondary BAs | 1.811(1.186, 3.088) | 1.417(1.036, 2.621) | 1.722(1.095, 2.292) | 0.128 |
| Unconjugated BAs (μmol/L) | 1.040(0.518, 2.066) | 0.795(0.361, 1.633) | 0.943(0.494, 1.433) | 0.443 |
| Conjugated BAs (μmol/L) | 1.686(1.016, 2.940) ^**##^ | 1.021(0.566, 2.107) | 1.007(0.710, 1.695) | <0.001 |
| Conjugated/Unconjugated BAs | 1.754(0.829, 3.475) ^*^ | 0.907(0.527, 2.331) | 1.108(0.701, 2.256) | 0.028 |
| Neurotoxic BAs (μmol/L) | 1.210(0.726, 2.099) ^##^ | 0.774(0.431, 1.823) | 0.702(0.468, 1.406) | 0.004 |
| Neuroprotective/Neurotoxic BAs (μmol/L) | 0.271(0.112, 0.582) ^**^ | 0.143(0.075, 0.325) | 0.226(0.125, 0.491) | 0.010 |
| %Conjugated CAs | 0.085(0.054, 0.134) ^#^ | 0.081(0.055, 0.112) | 0.058(0.040, 0.103) | 0.022 |
| %Conjugated CDCAs | 0.298(0.211, 0.435) | 0.227(0.134, 0.369) | 0.307(0.201, 0.370) | 0.063 |
| %Conjugated UDCAs | 0.076(0.032, 0.131) ^****^ | 0.033(0.017, 0.058) ^&&^ | 0.059(0.036, 0.081) | <0.001 |
| %Conjugated LCAs | 0.002(0.001, 0.007) ^***^ | 0.007(0.003, 0.010) ^&&&^ | 0.007(0.001, 0.010) | <0.001 |
| %Conjugated DCAs | 0.062(0.014, 0.106) ^***^ | 0.112(0.050, 0.142) | 0.085(0.039, 0.141) | 0.001 |
| %G-Conjugated BAs | 0.555(0.394, 0.686) | 0.428(0.309, 0.630) | 0.476(0.357, 0.634) | 0.078 |
| %T-Conjugated BAs | 0.057(0.027, 0.103) | 0.043(0.028, 0.068) | 0.056(0.033, 0.086) | 0.077 |
| %Unconjugated BAs | 0.363(0.224, 0.547) ^*^ | 0.524(0.300, 0.655) | 0.475(0.308, 0.588) | 0.028 |
| %Conjugated BAs | 0.648(0.215, 1.261) | 0.636(0.264, 1.685) | 0.574(0.304, 1.267) | 0.994 |
| %Primary BAs | 0.645(0.545, 0.754) | 0.586(0.509, 0.724) | 0.633(0.523, 0.696) | 0.123 |
| %Secondary BAs | 0.355(0.246, 0.455) | 0.414(0.276, 0.491) | 0.367(0.304, 0.477) | 0.123 |
| %12-OH BAs | 0.329(0.208, 0.501) ^***^ | 0.466(0.386, 0.626) ^&^ | 0.394(0.255, 0.515) | 0.001 |
| %Non-12-OH BAs | 0.671(0.499, 0.793) ^***^ | 0.534(0.374, 0.615) ^&^ | 0.606(0.485, 0.745) | 0.001 |
| CA (Conj./Unconj. BAs) | 2.426(0.728, 6.064) | 1.479(1.041, 3.490) | 1.760(0.653, 5.074) | 0.309 |
| CDCA (Conj./Unconj. BAs) | 2.650(0.914, 6.338) | 1.314(0.655, 5.342) | 1.845(0.798, 5.417) | 0.232 |
| UDCA (Conj./Unconj. BAs) | 2.319(1.165, 5.506) | 1.590(0.874, 4.739) | 1.488(0.866, 4.644) | 0.372 |
| LCA (Conj./Unconj. BAs) | 2.000(1.092, 2.729) ^####^ | 1.750(1.153, 2.813) ^&&&&^ | 1.000(0.714, 1.688) | <0.001 |
| DCA (Conj./Unconj. BAs) | 0.875(0.442, 2.000) ^#^ | 0.492(0.294, 1.060) | 0.585(0.331, 1.000) | 0.010 |
| GCA/TCA | 7.604(4.316, 13.430) | 7.976(4.809, 12.750) | 6.956(5.029, 13.470) | 0.918 |
| GCDCA/TCDCA | 8.540(5.332, 16.440) ^*#^ | 11.080(7.974, 18.480) | 11.320(7.961, 18.490) | 0.009 |
| GUDCA/TUDCA | 33.140(17.150, 50.750) | 32.420(18.750, 53.000) | 25.530(17.170, 42.630) | 0.232 |
| GLCA/TLCA | 1.333(1.000, 4.750) ^****##^ | 5.000(2.875, 7.222) | 3.000(2.000, 4.571) | <0.001 |
| GDCA/TDCA | 5.230(3.000, 9.383) ^****####^ | 9.063(6.035, 14.940) | 9.000(6.023, 15.220) | <0.001 |
| CA/CDCA | 0.296(0.167, 0.754) | 0.540(0.156, 0.860) | 0.286(0.125, 0.677) | 0.075 |
| LCA/DCA | 0.027(0.014, 0.092) | 0.023(0.010, 0.035) ^&^ | 0.031(0.021, 0.056) | 0.027 |
| LCA/UDCA | 0.040(0.012, 0.216) ^***###^ | 0.174(0.062, 0.794) | 0.172(0.057, 0.750) | <0.001 |
| %CDCAs | 0.473(0.361, 0.584) | 0.437(0.322, 0.548) | 0.485(0.387, 0.554) | 0.200 |
| %CAs | 0.148(0.101, 0.230) ^#^ | 0.151(0.102, 0.222) | 0.117(0.079, 0.178) | 0.017 |
| %DCAs | 0.166(0.046, 0.291) ^****####^ | 0.296(0.160, 0.424) | 0.297(0.159, 0.425) | <0.001 |
| %UDCAs | 0.108(0.049, 0.205) ^***###^ | 0.047(0.029, 0.123) | 0.047(0.029, 0.123) | <0.001 |
| %LCAs | 0.004(0.002, 0.011) ^***###^ | 0.011(0.005, 0.016) | 0.011(0.004, 0.016) | <0.001 |

**Notes:** NMOSD, neuromyelitis optica spectrum disorder; MS, multiple sclerosis; Data are presented as median (25th percentile, 75th percentile). *P*-values (last column) were derived from the Kruskal–Wallis H test for intergroup comparisons. ^*^*P* < 0.05, ^**^*P* < 0.01, ^***^*P* < 0.001 for NMOSD vs HC. ^#^*P* < 0.05, ^##^*P* < 0.01, ^###^*P* < 0.001 for NMOSD vs MS. ^&^*P* < 0.05, ^&&^*P* < 0.01, ^&&&^*P* < 0.001 for MS vs H

**Table S2. Diagnostic performance of differential bile acids for discriminating NMOSD from MS using ROC curve analysis**

| **Indicators** | **AUC** | **95% confidence interval** | ***P*-value** |
| --- | --- | --- | --- |
| GCDCA | 0.627 | 0.531-0.722 | 0.010 |
| TCDCA | 0.684 | 0.559-0.768 | <0.001 |
| UDCA | 0.663 | 0.576-0.749 | 0.001 |
| GUDCA | 0.720 | 0.639-0.801 | <0.001 |
| TUDCA | 0.732 | 0.655-0.808 | <0.001 |
| DCA | 0.607 | 0.517-0.698 | 0.030 |
| LCA | 0.602 | 0.505-.0699 | 0.038 |
| GLCA | 0.699 | 0.617-0.781 | <0.001 |
| TBA | 0.619 | 0.518-0.720 | 0.015 |
| %TCDCA | 0.649 | 0.56-0.736 | 0.003 |
| %DCA | 0.723 | 0.641-0.805 | <0.001 |
| %GDCA | 0.701 | 0.617-0.784 | <0.001 |
| %UDCA | 0.607 | 0.518-0.696 | 0.030 |
| %GUDCA | 0.695 | 0.6113-0.778 | <0.001 |
| %TUDCA | 0.669 | 0.586-0.751 | 0.001 |
| %LCA | 0.682 | 0.599-0.766 | <0.001 |
| %GLCA | 0.748 | 0.673-0.823 | <0.001 |
| CDCAs | 0.628 | 0.530-0.725 | 0.009 |
| UDCAs | 0.717 | 0.636-0.798 | <0.001 |
| LCAs | 0.631 | 0.539-0.722 | 0.008 |
| Non-12-OH BAs | 0.666 | 0.571-0.761 | 0.001 |
| 12-OH/Non-12-OH BAs | 0.693 | 0.609-0.776 | <0.001 |
| Conjugated BAs | 0.654 | 0.546-0.743 | 0.003 |
| Conjugated CDCAs | 0.641 | 0.548-0.734 | 0.004 |
| Conjugated UDCAs | 0.724 | 0.644-0.804 | <0.001 |
| Conjugated LCAs | 0.631 | 0.539-0.722 | 0.008 |
| G-Conjugated BAs | 0.631 | 0.631-0.731 | 0.008 |
| T-Conjugated BAs | 0.663 | 0.576-0.751 | 0.001 |
| Primary BAs | 0.626 | 0.528-0.725 | 0.010 |
| Conjugated/Unconjugated BAs | 0.615 | 0.521-0.708 | 0.020 |
| Neuroprotective/Neurotoxic BAs | 0.644 | 0.558-0.730 | 0.004 |
| %Conjugated UDCAs | 0.697 | 0.615-0.780 | <0.001 |
| %Conjugated LCAs | 0.703 | 0.622-0.784 | <0.001 |
| %Conjugated DCAs | 0.685 | 0.600-0.769 | <0.001 |
| %Unconjugated BAs | 0.615 | 0.521-0.708 | 0.020 |
| %12-OH BAs | 0.692 | 0.608-0.776 | <0.001 |
| %Non-12-OH BAs | 0.692 | 0.608-0.776 | <0.001 |
| GCDCA/TCDCA | 0.623 | 0.536-0.709 | 0.013 |
| GLCA/TLCA | 0.724 | 0.643-0.804 | <0.001 |
| GDCA/TDCA | 0.715 | 0.635-0.794 | <0.001 |
| LCA/UDCA | 0.689 | 0.605-0.772 | <0.001 |
| %DCAs | 0.720 | 0.640-0.800 | <0.001 |
| %UDCAs | 0.691 | 0.607-0.774 | <0.001 |
| %LCAs | 0.693 | 0.610-0.775 | <0.001 |

**Notes:** NMOSD, neuromyelitis optica spectrum disorder; MS, multiple sclerosis; ROC, receiver operating characteristic; AUC, area under the curve values.

**Table S3. Comparison of Baseline Clinical Characteristics among Patients with Overall Sample Set, Training Set and Test Set**

| **Characteristic** | **Overall Sample Set** | **Training Set** | **Test Set** | ***P*-value** |
| --- | --- | --- | --- | --- |
| NMOSD | 112(69.1%) | 90(69.8%) | 22(66.7%) | 0.72 |
| MS | 50(30.9%) | 39(30.2%) | 11(33.3%) | 0.72 |
| Age (years) | 36±11 | 37±11 | 35±10 | 0.898 |
| Female (%) | 134 (82.72%) | 107 (82.95%) | 27 (81.82%) | 0.960 |
| EDSS score | 3.75(3.00, 5.00) | 4.00(3.00, 5.00) | 3.50(2.75, 4.75) | 0.971 |
| Disease duration (years) | 5.15(1.50, 13.80) | 4.90(1.50, 12.90) | 7.6(2.00, 21.25) | 0.229 |
| LESCL (%) | 99(61.11%) | 77(59.59%) | 22(66.67%) | 0.764 |
| ALT (U/L) | 25(21, 33) | 25(21, 34) | 27(20, 32) | 0.995 |
| AST (U/L) | 22(18, 28) | 21(18, 29) | 24(17, 28) | 0.999 |
| Albumin (g/L) | 44.3(43.5, 45.0) | 44.4(43.6, 45) | 44.0(43.4, 44.8) | 0.435 |
| γ-GGT (U/L) | 30(21, 41) | 30(23, 45) | 30(18, 39) | 0.639 |
| APL (U/L) | 78(66, 91) | 78(66, 92) | 83(63, 92) | 0.984 |
| Dyslipidemia (%) | 16(9.88%) | 13(10.08%) | 3(9.09%) | 0.986 |
| Systolic pressure (mmHg) | 123.62±11.33 | 123.87±11.69 | 122.64±9.89 | 0.857 |
| Diastolic pressure (mmHg) | 78.16±11.21 | 78.21±11.33 | 77.97±10.91 | 0.994 |
| History of diabetes (%) | 6(3.70%) | 5(3.88%) | 1(3.03%) | 0.973 |

**Notes:** EDSS, Expanded Disability Status Scale; LESCL, longitudinally extensive spinal cord lesion; ON, optic neuritis; APS, acute phase syndrome; ALT, alanine aminotransferase; AST, aspartate aminotransferase; γ-GGT, gamma-glutamyl transferase; ALP, alkaline phosphatase. *P*-values (last column) were derived from the Kruskal–Wallis H test for intergroup comparisons.

**Table S4. Statistical Significance of Pairwise Model Comparisons (AUC).**

| **Model Comparison** | **Raw *P*-Value** | **Adjusted *P*-Value (Holm-Bonferroni)** | **Significant (α=0.05)** |
| --- | --- | --- | --- |
| Logistic Regression (Stable Features) vs. Random Forest | <0.0001 | <0.0001 | Yes |
| Logistic Regression (Stable Features) vs. SVM | <0.0001 | <0.0001 | Yes |
| Logistic Regression (Stable Features) vs. XGBoost | <0.0001 | <0.0001 | Yes |
| Logistic Regression (Stable Features) vs. LASSO + Logistic | >1.000 | 1.000 | No |
| Random Forest vs. SVM | <0.0001 | <0.0001 | Yes |
| Random Forest vs. XGBoost | <0.0001 | <0.0001 | Yes |
| Random Forest vs. LASSO + Logistic | <0.0001 | <0.0001 | Yes |
| SVM vs. XGBoost | <0.0001 | <0.0001 | Yes |
| SVM vs. LASSO + Logistic | <0.0001 | <0.0001 | Yes |
| XGBoost vs. LASSO + Logistic | <0.0001 | <0.0001 | Yes |

**Notes:** Pairwise statistical comparisons were conducted using t-tests on AUC differences derived from 1000 bootstrap samples, with subsequent Holm-Bonferroni correction for multiple comparisons.

**Table S5. Model Performance Comparison (AUC) Based on Repeated Cross-Validation**

| **Model** | **Mean** | **Std** | **Median** | **Q1** | **Q3** | **Min** | **Max** | **CV** |
| --- | --- | --- | --- | --- | --- | --- | --- | --- |
| LASSO+Logistic (LASSO Features) | 0.863 | 0.055 | 0.872 | 0.821 | 0.908 | 0.722 | 0.965 | 0.064 |
| Logistic (Stable Features) | 0.856 | 0.056 | 0.854 | 0.826 | 0.894 | 0.736 | 0.965 | 0.066 |
| SVM | 0.851 | 0.059 | 0.861 | 0.806 | 0.894 | 0.729 | 0.958 | 0.069 |
| Random Forest | 0.817 | 0.064 | 0.811 | 0.776 | 0.869 | 0.677 | 0.963 | 0.078 |
| XGBoost | 0.760 | 0.084 | 0.757 | 0.703 | 0.817 | 0.583 | 0.949 | 0.110 |

**Notes:** Std, Standard Deviation; Q1, First Quartile; Q3, Third Quartile; Min, Minimum; Max, Maximum; CV, Coefficient of Variation.

**Table S6. Bootstrap Validation Performance of Predictive Models**

| **Model** | **AUC (95% CI)** | **Accuracy (95% CI)** | **Balanced Accuracy (95% CI)** | **Sensitivity (95% CI)** | **Specificity (95% CI)** |
| --- | --- | --- | --- | --- | --- |
| Logistic (Stable Features) | 0.874 (0.735–0.975) | 0.789 (0.636–0.909) | 0.820 (0.673–0.935) | 0.902 (0.667–1.000) | 0.738 (0.545–0.909) |
| Random Forest | 0.825 (0.660–0.959) | 0.785 (0.636–0.909) | 0.703 (0.542–0.864) | 0.491 (0.167–0.818) | 0.915 (0.792–1.000) |
| SVM | 0.883 (0.739–0.980) | 0.759 (0.606–0.909) | 0.797 (0.639–0.921) | 0.896 (0.667–1.000) | 0.697 (0.500–0.880) |
| XGBoost | 0.717 (0.504–0.883) | 0.754 (0.606–0.880) | 0.683 (0.512–0.850) | 0.497 (0.167–0.818) | 0.868 (0.708–1.000) |
| LASSO+Logistic (LASSO Features) | 0.874 (0.736–0.978) | 0.849 (0.697–0.970) | 0.864 (0.722–0.975) | 0.902 (0.667–1.000) | 0.826 (0.666–0.960) |

**Note:** Performance metrics are reported as mean values with 95% confidence intervals (CI) derived from bootstrap validation. AUC: Area Under the Receiver Operating Characteristic Curve.

**Table S7. Clinical Utility Assessment (Using Logistic Regression (Stable Features) as Baseline)**

| **Comparison Model** | **Total NRI** | **Event NRI** | **Non-event NRI** | **Total IDI** |
| --- | --- | --- | --- | --- |
| Random Forest | -0.457 | -0.500 | 0.044 | -0.096 |
| SVM | -0.126 | -0.300 | 0.174 | 0.009 |
| XGBoost | 0.004 | -0.300 | 0.304 | -0.089 |
| LASSO+Logistic | -0.244 | -0.200 | -0.044 | -0.013 |

**Notes:** NRI, Net Reclassification Index; IDI, Integrated Discrimination Improvement. A negative value indicates performance degradation compared to the baseline model. Event NRI indicates the improvement in correct classification for NMOSD patients.

**Table S8. Comparison of Baseline Clinical Characteristics among Patients with AQP4-IgG (-) and AQP4-IgG (-)**

| **Characteristic** | **AQP4-IgG (-) (n=32)** | **AQP4-IgG (+) (n=80)** | ***P*-value** |
| --- | --- | --- | --- |
| Age (years) | 35±10 | 37±10 | 0.225 |
| Female (%) | 20(62.50%) | 56(70%) | 0.443 |
| EDSS score | 4.5(3.5-5) | 4.5(3.5-5.5) | 0.714 |
| Disease duration(year) | 4.00(1.23–8.43) | 6.30(1.63–17.00) | 0.258 |
| LESCL (%) | 27(84.38%) | 67(83.75%) | 0.935 |
| ON (%) | 21(65.63%) | 50(62.50) | 0.757 |
| APS (%) | 2(6.25%) | 24(30.00%) | 0.479 |
| ALT (U/L) | 25(17, 38) | 25(19, 33) | 0.652 |
| AST (U/L) | 22(15, 36) | 22(16, 31) | 0.721 |
| Albumin (g/L) | 44.3(43.8, 45.2) | 44.2(43.5, 45.0) | 0.325 |
| γ-GGT (U/L) | 29(17, 49) | 30(20, 42) | 0.789 |
| ALP (U/L) | 77(59, 98) | 78(64, 95) | 0.715 |
| Dyslipidemia (%) | 3(9.37%) | 9(11.25%) | 0.771 |
| Systolic pressure (mmHg) | 123.19±10.49 | 122.81±10.88 | 0.912 |
| Diastolic pressure (mmHg) | 79.19±11.70 | 77.23±10.53 | 0.385 |
| History of diabetes (%) | 1(3.13%) | 4(5.00%) | 0.664 |

**Notes:** EDSS, Expanded Disability Status Scale; LESCL, longitudinally extensive spinal cord lesion; ON, optic neuritis; APS, acute phase syndrome; ALT, alanine aminotransferase; AST, aspartate aminotransferase; γ-GGT, gamma-glutamyl transferase; ALP, alkaline phosphatase. Data were analyzed using the Mann–Whitney U test.

**Table S9. Comparison of Bile Acid Metabolic Profiles with AQP4-IgG (-) and AQP4-IgG (+)**

| **Bile acid** | **AQP4-IgG (-) (n=32)** | **AQP4-IgG (+) (n=80)** | **MS(n=50)** | ***P*-value** |
| --- | --- | --- | --- | --- |
| CDCA (μmol/L) | 0.486(0.229, 0.922) | 0.349(0.158, 0.873) | 0.259(0.084, 0.741) | 0.224 |
| GCDCA (μmol/L) | 0.819(0.446, 1.606) ^#^ | 0.654(0.434, 1.295) ^&^ | 0.507(0.232, 0.984) | 0.004 |
| TCDCA (μmol/L) | 0.105(0.038, 0.204) ^##^ | 0.082(0.037, 0.206) ^&&^ | 0.047(0.019, 0.078) | 0.001 |
| CA (μmol/L) | 0.088(0.037, 0.241) | 0.092(0.045, 0.431) | 0.098(0.036, 0.289) | 0.698 |
| GCA (μmol/L) | 0.486(0.229, 0.922) | 0.349(0.158, 0.873) | 0.130(0.074, 0.337) | 0.131 |
| TCA (μmol/L) | 0.026(0.013, 0.071) | 0.024(0.011, 0.055) | 0.018(0.010, 0.037) | 0.153 |
| UDCA (μmol/L) | 0.063(0.035, 0.230) ^#^ | 0.107(0.021, 0.261) ^&&^ | 0.150(0.029, 0.160) | 0.004 |
| GUDCA (μmol/L) | 0.182(0.077, 0.560) ^##^ | 0.188(0.088, 0.486) ^&&&&^ | 0.077(0.043, 0.174) | <0.001 |
| TUDCA (μmol/L) | 0.006(0.002, 0.023) ^###^ | 0.006(0.002, 0.015) ^&&&&^ | 0.002(0.001, 0.005) | <0.001 |
| DCA (μmol/L) | 0.005(0.001, 0.032) ^****####^ | 0.370(0.191, 0.729) | 0.268(0.142, 0.648) | <0.001 |
| GDCA (μmol/L) | 0.004(0.001, 0.019) ^****####^ | 0.240(0.114, 0.398) | 0.159(0.072, 0.365) | <0.001 |
| TDCA (μmol/L) | 0.001(0.001, 0.004) ^****####^ | 0.032(0.018, 0.067) | 0.021(0.008, 0.050) | <0.001 |
| LCA (μmol/L) | 0.001(0.001, 0.002) ^****####^ | 0.007(0.003, 0.013) | 0.007(0.003, 0.017) | <0.001 |
| GLCA (μmol/L) | 0.001(0.001, 0.003) ^****####^ | 0.006(0.002, 0.017) | 0.010(0.004, 0.021) | <0.001 |
| TLCA (μmol/L) | 0.001(0.001, 0.002) ^****^ | 0.004(0.001, 0.006) | 0.002(0.001, 0.003) | 0.001 |
| TBA (μmol/L) | 2.817(1.489, 5.319) | 3.518(2.059, 5.626) ^&&^ | 1.961(1.210, 4.083) | 0.013 |
| %CDCA | 0.186(0.095, 0.279) ^*^ | 0.105(0.049, 0.215) | 0.146(0.060, 0.284) | 0.039 |
| %GCDCA | 0.331(0.224, 0.470) ^*##^ | 0.239(0.137, 0.349) | 0.211(0.121, 0.327) | 0.007 |
| %TCDCA | 0.038(0.016, 0.094) ^###^ | 0.027(0.013, 0.051) | 0.019(0.009, 0.034) | 0.001 |
| %CA | 0.037(0.021, 0.068) | 0.032(0.014, 0.138) | 0.046(0.029, 0.091) | 0.207 |
| %GCA | 0.096(0.050, 0.131) | 0.068(0.042, 0.102) | 0.071(0.046, 0.093) | 0.147 |
| %TCA | 0.015(0.005, 0.025) | 0.007(0.004, 0.018) | 0.009(0.005, 0.013) | 0.200 |
| %DCA | 0.002(<0.001, 0.018) ^****####^ | 0.125(0.059, 0.202) | 0.171(0.100, 0.295) | <0.001 |
| %GDCA | 0.002(<0.001, 0.011) ^****####^ | 0.073(0.041, 0.098) | 0.101(0.047, 0.129) | <0.001 |
| %TDCA | 0.001(<0.001, 0.003) ^****####^ | 0.009(0.005, 0.021) | 0.011(0.005, 0.017) | <0.001 |
| %UDCA | 0.020(0.009, 0.134) | 0.029(0.010, 0.068) | 0.021(0.007, 0.050) | 0.081 |
| %GUDCA | 0.090(0.033, 0.165) ^###^ | 0.070(0.028, 0.114) ^&&^ | 0.031(0.017, 0.056) | <0.001 |
| %TUDCA | 0.003(0.001, 0.007) ^###^ | 0.002(0.001, 0.005) ^&^ | 0.001(0.001, 0.002) | 0.001 |
| %LCA | <0.001(<0.001, 0.002) ^****####^ | 0.002(0.001, 0.004) | 0.003(0.002, 0.006) | <0.001 |
| %GLCA | <0.001(<0.001, 0.001) ^####^ | 0.002(0.001, 0.007) ^&&&&^ | 0.006(0.002, 0.009) | <0.001 |
| %TLCA | <0.001(<0.001, 0.001) ^#^ | 0.001(<0.001, 0.002) | 0.001(0.001, 0.002) | 0.018 |
| CDCAs (μmol/L) | 1.465(1.008, 3.770) | 1.466(0.796, 2.657) | 0.925(0.473, 1.653) | 0.469 |
| CAs (μmol/L) | 0.418(0.222, 0.741) | 0.482(0.231, 0.887) | 0.318(0.137, 0.856) | 0.184 |
| DCAs (μmol/L) | 0.009(0.003, 0.059) ^####^ | 0.752(0.412, 0.122) ^&&&&^ | 0.495(0.263, 1.250) | <0.001 |
| UDCAs (μmol/L) | 0.261(0.139, 1.002) ^##^ | 0.317(0.126, 0.823) ^&&&&^ | 0.123(0.069, 0.306) | <0.001 |
| LCAs (μmol/L) | 0.004(0.003, 0.007) ^****####^ | 0.018(0.010, 0.034) | 0.021(0.009, 0.037) | <0.001 |
| 12-OH BAs (μmol/L) | 0.438(0.229, 0.806) ^****##^ | 1.296(0.774, 2.180) | 0.832(0.398, 2.441) | <0.001 |
| Non-12-OH BAs (μmol/L) | 2.246(1.128, 4.570) ^##^ | 1.920(1.129, 3.641) ^&&^ | 1.159(0.542, 1.992) | 0.003 |
| 12-OH/Non-12-OH BAs | 0.190(0.148, 0.342) ^****####^ | 0.666(0.392, 1.276) | 0.872(0.628, 1.675) | <0.001 |
| Conjugated CAs (μmol/L) | 0.269(0.124, 0.457) | 0.250(0.139, 0.500) | 0.150(0.088, 0.426) | 0.118 |
| Conjugated CDCAs (μmol/L) | 0.984(0.534, 1.810) ^#^ | 0.754(0.516, 1.375) ^&^ | 0.565(0.253, 1.075) | 0.012 |
| Conjugated DCAs (μmol/L) | 0.005(0.002, 0.030) ^****####^ | 0.564(0.155, 0.468) | 0.186(0.083, 0.413) | <0.001 |
| Conjugated UDCAs (μmol/L) | 0.193(0.092, 0.622) ^##^ | 0.196(0.093, 0.500) ^&&&&^ | 0.076(0.044, 0.179) | <0.001 |
| Conjugated LCAs (μmol/L) | 0.002(0.002, 0.004) ^****####^ | 0.009(0.006, 0.019) | 0.012(0.005, 0.024) | <0.001 |
| G-Conjugated BAs (μmol/L) | 1.576(0.967, 2.543) | 1.385(0.870, 2.760) ^&^ | 0.926(0.470, 1.898) | 0.029 |
| T-Conjugated BAs (μmol/L) | 0.152(0.069, 0.309) | 0.155(0.080, 0.367) ^&&^ | 0.090(0.045, 0.154) | 0.004 |
| G/T-Conjugated BAs | 9.181(5.095, 20.00) | 8.967(5.474, 15.82) | 10.580(7.614, 15.970) | 0.347 |
| Primary BAs (μmol/L) | 1.889(1.246, 5.172) | 2.148(1.236, 3.735) ^&^ | 1.305(0.694, 2.334) | 0.037 |
| Secondary BAs (μmol/L) | 0.230(0.189, 1.011) ^****#^ | 1.304(0.867, 2.016) ^&&^ | 0.706(0.411, 1.424) | <0.001 |
| Primary/Secondary BAs | 3.429(1.599, 16.03) ^****####^ | 1.574(0.976, 2.526) | 1.417(1.036, 2.621) | <0.001 |
| Unconjugated BAs (μmol/L) | 0.757(0.374, 1.674) | 1.062(0.627, 2.268) | 0.795(0.361, 1.633) | 0.072 |
| Conjugated BAs (μmol/L) | 1.844(1.109, 2.643) | 1.659(0.972, 3.012) ^&^ | 1.021(0.566, 2.107) | 0.013 |
| Conjugated/Unconjugated BAs | 2.670(0.925, 4.219) ^#^ | 1.589(0.700, 3.064) | 0.907(0.527, 2.331) | 0.017 |
| Neurotoxic BAs (μmol/L) | 1.051(0.575, 2.005) | 1.270(0.726, 2.229) | 0.774(0.431, 1.823) | 0.097 |
| Neuroprotective/Neurotoxic BAs (μmol/L) | 0.199(0.085, 1.044) | 0.288(0.123, 0.478) ^&^ | 0.143(0.075, 0.325) | 0.014 |
| %Conjugated CAs | 0.105(0.058, 0.164) | 0.077(0.046, 0.121) | 0.081(0.055, 0.112) | 0.073 |
| %Conjugated CDCAs | 0.390(0.250, 0.561) ^**###^ | 0.272(0.159, 0.394) | 0.227(0.134, 0.369) | 0.001 |
| %Conjugated UDCAs | 0.095(0.036, 0.171) ^###^ | 0.071(0.030, 0.119) ^&&^ | 0.033(0.017, 0.058) | <0.001 |
| %Conjugated LCAs | 0.001(<0.001, 0.002) ^****####^ | 0.002(0.001, 0.008) | 0.007(0.003, 0.010) | <0.001 |
| %Conjugated DCAs | 0.003(0.001, 0.013) ^****####^ | 0.088(0.046, 0.120) | 0.112(0.050, 0.142) | <0.001 |
| %G-Conjugated BAs | 0.581(0.443, 0701) | 0.547(0.359, 0.678) | 0.428(0.309, 0.630) | 0.099 |
| %T-Conjugated BAs | 0.059(0.028, 0.126) | 0.056(0.026, 0.101) | 0.043(0.028, 0.068) | 0.070 |
| %Unconjugated BAs | 0.273(0.0.193, 0.519) ^#^ | 0.386(0.246, 0.588) | 0.524(0.300, 0.655) | 0.017 |
| %Conjugated BAs | 1.027(0.362, 2.189) | 0.5829(0.187, 1.170) | 0.636(0.264, 1.685) | 0.126 |
| %Primary BAs | 0.773(0.615, 0.941) ^****####^ | 0.612(0.494, 0.716) | 0.586(0.509, 0.724) | <0.001 |
| %Secondary BAs | 0.227(0.059, 0.385) ^****####^ | 0.389(0.284, 0.506) | 0.414(0.276, 0.491) | <0.001 |
| %12-OH BAs | 0.160(0.129, 0.255) ^****####^ | 0.400(0.281, 0.561) | 0.466(0.386, 0.626) | <0.001 |
| %Non-12-OH BAs | 0.841(0.745, 0.871) ^****####^ | 0.600(0.439, 0.719) | 0.534(0.374, 0.615) | <0.001 |
| CA (Conj./Unconj. BAs) | 3.756(1.099, 5.143) | 0.221(0.056, 0.278) | 1.479(1.041, 3.490) | 0.203 |
| CDCA (Conj./Unconj. BAs) | 2.660(0.952, 5.800) | 2.650(0.914, 8.154) | 1.314(0.655, 5.342) | 0.243 |
| UDCA (Conj./Unconj. BAs) | 2.755(1.256, 4.436) | 2.152(1.145, 5.991) | 1.590(0.874, 4.739) | 0.580 |
| LCA (Conj./Unconj. BAs) | 2.000(1.500, 2.000) | 1.529(1.000, 3.000) | 1.750(1.153, 2.813) | 0.546 |
| DCA (Conj./Unconj. BAs) | 1.859(0.774, 2.000) ^**###^ | 0.738(0.370, 1.296) | 0.492(0.294, 1.060) | <0.001 |
| GCA/TCA | 7.360(3.784, 14.54) | 7.879(4.514, 12.480) | 7.976(4.809, 12.750) | 0.938 |
| GCDCA/TCDCA | 8.382(4.133, 17.100) | 8.803(5.420, 16.210) | 11.080(7.974, 18.480) | 0.056 |
| GUDCA/TUDCA | 35.500(15.090, 56.000) | 31.800(19.280, 49.150) | 32.420(18.750, 53.000) | 0.980 |
| GLCA/TLCA | 1.000(1.000, 1.000) ^***####^ | 2.528(0.893, 5.773) ^&&^ | 5.000(2.875, 7.222) | <0.001 |
| GDCA/TDCA | 1.979(1.000, 5.083) ^****####^ | 6.127(4.025, 11.290) ^&^ | 9.063(6.035, 14.940) | <0.001 |
| CA/CDCA | 0.225(0.136, 0.380) ^*#^ | 0.420(0.192, 0.917) | 0.540(0.156, 0.860) | 0.008 |
| LCA/DCA | 0.310(0.063, 1.000) ^****####^ | 0.017(0.011, 0.032) | 0.023(0.010, 0.035) | <0.001 |
| LCA/UDCA | 0.018(0.005, 0.037) ^**####^ | 0.085(0.017, 0.333) ^&^ | 0.174(0.062, 0.794) | <0.001 |
| %CDCAs | 0.587(0.468, 0.727) ^****####^ | 0.431(0.341, 0.516) | 0.437(0.322, 0.548) | <0.001 |
| %CAs | 0.152(0.120, 0.216) | 0.145(0.095, 0.235) | 0.151(0.102, 0.222) | 0.775 |
| %DCAs | 0.005(0.001, 0.025) ^****####^ | 0.217(0.130, 0.359) | 0.296(0.160, 0.424) | <0.001 |
| %UDCAs | 0.114(0.049, 0.377) ^##^ | 0.106(0.050, 0.173) ^&&^ | 0.047(0.029, 0.123) | 0.331 |
| %LCAs | 0.001(0.001, 0.004) ^****####^ | 0.005(0.003, 0.013) | 0.011(0.005, 0.016) | <0.001 |

**Notes:** AQP4-IgG, anti–aquaporin-4 immunoglobulin G. Data are presented as median (25th percentile, 75th percentile). *P*-values (last column) were derived from the Kruskal–Wallis H test for intergroup comparisons. ^*^*P* < 0.05, ^**^*P* < 0.01, ^***^*P* < 0.001 for AQP4-IgG (-) vs MS. ^#^*P* < 0.05, ^##^*P* < 0.01, ^###^*P* < 0.001 for AQP4-IgG (-) vs AQP4-IgG (+). ^&^*P* < 0.05, ^&&^*P* < 0.01, ^&&&^*P* < 0.001 for AQP4-IgG (+) vs MS.

**Table S10. Diagnostic performance of differential bile acids for discriminating AQP4-IgG (-) from MS using ROC curve analysis.**

| **Indicator** | **Area** | **95% confidence interval** | ***P*-value** |
| --- | --- | --- | --- |
| GCDCA | 0.673 | 0.556-0.790 | 0.006 |
| TCDCA | 0.713 | 0.593-0.832 | 0.001 |
| UDCA | 0.658 | 0.536-0.781 | 0.016 |
| GUDCA | 0.711 | 0.595-0.827 | 0.001 |
| TUDCA | 0.740 | 0.625-0.855 | <0.001 |
| DCA | 0.965 | 0.924-1.000 | <0.001 |
| GDCA | 0.943 | 0.896-0.990 | <0.001 |
| TLCA | 0.851 | 0.765-0.936 | <0.001 |
| LCA | 0.862 | 0.782-0.941 | <0.001 |
| GLCA | 0.953 | 0.908-0.998 | <0.001 |
| TLCA | 0.684 | 0.555-0.814 | 0.005 |
| %GCDCA | 0.704 | 0.590-0.819 | 0.002 |
| %DCA | 0.964 | 0.929-1.000 | <0.001 |
| %GDCA | 0.939 | 0.883-0.996 | <0.001 |
| %TDCA | 0.865 | 0.768-0.963 | <0.001 |
| %GUDCA | 0.744 | 0.633-0.854 | <0.001 |
| %TUDCA | 0.733 | 0.617-0.850 | 0.001 |
| %LCA | 0.857 | 0.769-0.944 | <0.001 |
| %GLCA | 0.931 | 0.878-0.983 | <0.001 |
| %TLCA | 0.682 | 0.553-0.811 | 0.006 |
| DCAs | 0.965 | 0.923-1.000 | <0.001 |
| UDCAs | 0.702 | 0.5845-0.820 | 0.002 |
| LCAs | 0.904 | 0.841-0.967 | <0.001 |
| 12-OH BAs | 0.681 | 0.566-0.796 | 0.006 |
| Non-12-OH BAs | 0.683 | 0.564-0.802 | 0.005 |
| 12-OH/Non-12-OH BAs | 0.923 | 0.865-0.981 | <0.001 |
| Conjugated CDCAs | 0.665 | 0.543-0.787 | 0.012 |
| Conjugated DCAs | 0.932 | 0.880-0.983 | <0.001 |
| Conjugated UDCAs | 0.718 | 0.603-0.833 | 0.001 |
| Conjugated LCAs | 0.901 | 0.837-0.965 | <0.001 |
| Secondary BAs | 0.689 | 0.566-0.813 | 0.004 |
| Primary/Secondary BAs | 0.768 | 0.664-0.873 | <0.001 |
| Conjugated/Unconjugated BAs | 0.684 | 0.564-0.804 | 0.005 |
| %Conjugated CDCAs | 0.741 | 0.631-0.852 | <0.001 |
| %Conjugated UDCAs | 0.753 | 0.644-0.861 | <0.001 |
| %Conjugated LCAs | 0.89 | 0.767-0.952 | <0.001 |
| %Conjugated DCAs | 0.929 | 0.866-0.993 | <0.001 |
| %Unconjugated BAs | 0.684 | 0.564-0.804 | 0.005 |
| %Primary BAs | 0.692 | 0.608-0.776 | <0.001 |
| %Secondary BAs | 0.692 | 0.608-0.776 | <0.001 |
| %12-OH BAs | 0.923 | 0.865-0.981 | <0.001 |
| Non-12-OH BAs | 0.923 | 0.865-0.981 | <0.001 |
| DCA (Conj./Unconj. BAs) | 0.750 | 0.642-0.858 | <0.001 |
| GLCA/TLCA | 0.908 | 0.843-0.974 | <0.001 |
| GDCA/TDCA | 0.866 | 0.778-0.854 | <0.001 |
| CA/CDCA | 0.690 | 0.575-0.805 | 0.004 |
| LCA/DCA | 0.892 | 0.815-0.969 | <0.001 |
| LCA/UDCA | 0.834 | 0.739-0.930 | <0.001 |
| %CDCAs | 0.786 | 0.689-0.882 | <0.001 |
| %DCAs | 0.964 | 0.927-1.000 | <0.001 |
| %UDCAs | 0.718 | 0.602-0.834 | 0.001 |
| %LCAs | 0.856 | 0.764-0.949 | <0.001 |

**Notes:** NMOSD, neuromyelitis optica spectrum disorder; MS, multiple sclerosis; ROC, receiver operating characteristic; AUC, area under the curve values.

**Table S11. R** **Bootstrap-based stability of the Youden-optimized cutoff and diagnostic indices for DCA (μmol/L)**

| **Biomarker** | **Bootstrap iterations** | **Cutoff (μmol/L) median (2.5–97.5%)** | **Sensitivity median (2.5–97.5%)** | **Specificity median (2.5–97.5%)** | **PPV median (2.5–97.5%)** | **NPV median (2.5–97.5%)** |
| --- | --- | --- | --- | --- | --- | --- |
| DCA | 200 | 0.068 (0.034–0.068) | 1.000 (0.938–1.000) | 0.920 (0.840–0.980) | 0.889 (0.800–0.970) | 1.000 (0.959–1.000) |

Notes: The optimal cutoff in each bootstrap sample was selected by maximizing Youden’s index. PPV and NPV are prevalence-dependent and were computed using the sample proportion in this study. These stability estimates are intended to quantify internal uncertainty; external validation is required before clinical implementation.

**Table S12. Correlation of Bile Acid Biomarkers with EDSS Scores and Spinal Cord MRI T2-Lesions in NMOSD and MS**

| **Indicator** | **NMOSD** | | | | **MS** | | | |  |
| --- | --- | --- | --- | --- | --- | --- | --- | --- | --- |
|  | **r(EDSS)** | ***P*-value** | **r(MRI)** | ***P*-value** | **r(EDSS)** | ***P*-value** | **r(MRI)** | ***P*-value** |  |
| CDCA | | 0.153 | 0.108 | 0.063 | 0.510 | 0.095 | 0.513 | -0.061 | 0.676 |
| GCDCA | | -0.091 | 0.338 | 0.027 | 0.774 | 0.114 | 0.429 | -0.071 | 0.625 |
| TCDCA | | -0.016 | 0.864 | 0.011 | 0.909 | 0.112 | 0.440 | -0.093 | 0.521 |
| CA | | 0.110 | 0.248 | -0.024 | 0.805 | 0.119 | 0.409 | -0.051 | 0.726 |
| GCA | | -0.019 | 0.843 | -0.040 | 0.679 | 0.194 | 0.178 | -0.077 | 0.595 |
| TCA | | 0.009 | 0.926 | -0.087 | 0.361 | 0.189 | 0.190 | -0.039 | 0.787 |
| DCA | | 0.257 | 0.006 | 0.041 | 0.671 | 0.397 | 0.004 | 0.009 | 0.948 |
| GDCA | | 0.080 | 0.402 | 0.023 | 0.813 | 0.261 | 0.067 | -0.010 | 0.945 |
| TDCA | | 0.030 | 0.752 | -0.012 | 0.904 | 0.341 | 0.015 | 0.009 | 0.952 |
| UDCA | | -0.190 | 0.045 | -0.040 | 0.678 | -0.160 | 0.268 | -0.046 | 0.751 |
| GUDCA | | -0.396 | <0.001 | -0.025 | 0.791 | 0.100 | 0.488 | -0.030 | 0.836 |
| TUDCA | | -0.315 | 0.001 | -0.040 | 0.674 | 0.244 | 0.088 | -0.047 | 0.744 |
| LCA | | 0.139 | 0.143 | 0.073 | 0.447 | 0.404 | 0.004 | -0.032 | 0.828 |
| GLCA | | 0.101 | 0.289 | 0.124 | 0.192 | 0.372 | 0.008 | -0.004 | 0.978 |
| TLCA | | 0.111 | 0.242 | 0.049 | 0.611 | 0.463 | 0.001 | -0.049 | 0.734 |
| TBA | | 0.046 | 0.630 | 0.088 | 0.357 | 0.183 | 0.205 | -0.056 | 0.699 |
| %CDCA | | 0.156 | 0.101 | 0.078 | 0.416 | -0.131 | 0.364 | -0.034 | 0.815 |
| %GCDCA | | -0.154 | 0.105 | -0.039 | 0.685 | -0.024 | 0.867 | -0.057 | 0.696 |
| %TCDCA | | -0.027 | 0.781 | -0.054 | 0.575 | 0.015 | 0.920 | 0.028 | 0.846 |
| %CA | | 0.151 | 0.111 | -0.078 | 0.414 | -0.073 | 0.612 | -0.037 | 0.798 |
| %GCA | | 0.012 | 0.902 | -0.142 | 0.137 | 0.084 | 0.560 | 0.083 | 0.567 |
| %TCA | | 0.052 | 0.589 | -0.116 | 0.224 | -0.002 | 0.987 | 0.082 | 0.573 |
| %DCA | | 0.242 | 0.010 | -0.005 | 0.955 | 0.170 | 0.238 | -0.042 | 0.773 |
| %GDCA | | 0.034 | 0.726 | -0.007 | 0.939 | 0.309 | 0.029 | 0.092 | 0.527 |
| %TDCA | | 0.031 | 0.742 | -0.046 | 0.630 | 0.241 | 0.092 | 0.121 | 0.403 |
| %UDCA | | -0.244 | 0.010 | -0.075 | 0.434 | -0.309 | 0.029 | 0.008 | 0.958 |
| %GUDCA | | -0.455 | <0.001 | -0.095 | 0.318 | -0.016 | 0.911 | 0.020 | 0.891 |
| %TUDCA | | -0.361 | <0.001 | -0.141 | 0.138 | -0.065 | 0.653 | -0.036 | 0.802 |
| %LCA | | 0.100 | 0.296 | 0.001 | 0.988 | 0.228 | 0.111 | 0.048 | 0.741 |
| %GLCA | | 0.063 | 0.510 | 0.034 | 0.719 | 0.209 | 0.144 | 0.071 | 0.623 |
| %TLCA | | 0.054 | 0.575 | -0.009 | 0.925 | 0.124 | 0.391 | 0.035 | 0.811 |
| CDCAs | | 0.033 | 0.729 | 0.085 | 0.373 | 0.092 | 0.523 | -0.070 | 0.628 |
| CAs | | 0.080 | 0.400 | -0.040 | 0.674 | 0.136 | 0.347 | -0.057 | 0.693 |
| DCAs | | 0.223 | 0.018 | 0.075 | 0.430 | 0.366 | 0.009 | 0.021 | 0.882 |
| UDCAs | | -0.327 | 0.000 | 0.001 | 0.996 | 0.028 | 0.846 | -0.023 | 0.876 |
| LCAs | | 0.112 | 0.241 | 0.081 | 0.395 | 0.432 | 0.002 | -0.011 | 0.939 |
| 12-OH BAs | | 0.181 | 0.056 | 0.028 | 0.767 | 0.288 | 0.042 | -0.016 | 0.913 |
| Non-12-OH BAs | | -0.051 | 0.594 | 0.070 | 0.463 | 0.117 | 0.418 | -0.059 | 0.687 |
| 12-OH/Non-12-OH BAs | | 0.229 | 0.015 | -0.049 | 0.610 | 0.183 | 0.202 | -0.052 | 0.720 |
| Conjugated CAs | | -0.007 | 0.939 | -0.050 | 0.597 | 0.179 | 0.214 | -0.057 | 0.693 |
| Conjugated CDCAs | | -0.072 | 0.448 | 0.027 | 0.775 | 0.113 | 0.435 | -0.076 | 0.599 |
| Conjugated DCAs | | 0.077 | 0.420 | 0.018 | 0.847 | 0.268 | 0.060 | -0.003 | 0.986 |
| Conjugated UDCAs | | -0.397 | <0.001 | -0.027 | 0.779 | 0.106 | 0.466 | -0.028 | 0.847 |
| Conjugated LCAs | | 0.081 | 0.395 | 0.076 | 0.424 | 0.396 | 0.005 | -0.012 | 0.932 |
| G-Conjugated BAs | | -0.138 | 0.147 | 0.025 | 0.792 | 0.196 | 0.174 | -0.082 | 0.573 |
| T-Conjugated BAs | | 0.002 | 0.981 | 0.000 | >0.999 | 0.232 | 0.104 | -0.063 | 0.663 |
| G/T-Conjugated BAs | | -0.135 | 0.156 | 0.039 | 0.684 | -0.090 | 0.536 | 0.014 | 0.922 |
| Primary BAs | | 0.064 | 0.505 | 0.061 | 0.523 | 0.107 | 0.461 | -0.053 | 0.714 |
| Secondary BAs | | -0.020 | 0.832 | 0.070 | 0.463 | 0.354 | 0.012 | -0.019 | 0.897 |
| Primary/Secondary BAs | | 0.065 | 0.499 | -0.036 | 0.704 | -0.196 | 0.173 | -0.011 | 0.942 |
| Unconjugated BAs | | 0.154 | 0.105 | 0.068 | 0.473 | 0.267 | 0.061 | -0.056 | 0.697 |
| Conjugated BAs | | -0.112 | 0.241 | 0.040 | 0.676 | 0.189 | 0.189 | -0.085 | 0.559 |
| Conjugated/Unconjugated BAs | | -0.206 | 0.030 | -0.046 | 0.631 | 0.025 | 0.861 | 0.012 | 0.934 |
| Neurotoxic BAs | | -0.048 | 0.618 | 0.032 | 0.735 | 0.186 | 0.196 | -0.078 | 0.588 |
| Neuroprotective/Neurotoxic BAs | | -0.276 | 0.003 | -0.035 | 0.711 | -0.186 | 0.196 | 0.034 | 0.817 |
| %Conjugated CAs | | 0.034 | 0.722 | -0.140 | 0.142 | 0.069 | 0.633 | 0.101 | 0.486 |
| %Conjugated CDCAs | | -0.135 | 0.157 | -0.039 | 0.680 | -0.030 | 0.836 | -0.039 | 0.787 |
| %Conjugated UDCAs | | -0.458 | <0.001 | -0.102 | 0.283 | -0.043 | 0.768 | 0.044 | 0.759 |
| %Conjugated LCAs | | 0.013 | 0.889 | -0.006 | 0.950 | 0.210 | 0.144 | 0.073 | 0.612 |
| %Conjugated DCAs | | 0.034 | 0.725 | -0.014 | 0.885 | 0.311 | 0.028 | 0.095 | 0.511 |
| %G-Conjugated BAs | | -0.239 | 0.011 | -0.027 | 0.777 | 0.016 | 0.913 | 0.009 | 0.953 |
| %T-Conjugated BAs | | 0.004 | 0.968 | -0.073 | 0.446 | 0.044 | 0.763 | 0.127 | 0.381 |
| %Unconjugated BAs | | 0.206 | 0.030 | 0.046 | 0.631 | -0.025 | 0.861 | -0.012 | 0.934 |
| %Conjugated BAs | | -0.166 | 0.081 | -0.085 | 0.372 | -0.241 | 0.092 | 0.063 | 0.664 |
| %Primary BAs | | 0.065 | 0.499 | -0.036 | 0.704 | -0.196 | 0.173 | -0.011 | 0.942 |
| %Secondary BAs | | -0.065 | 0.499 | 0.036 | 0.704 | 0.196 | 0.173 | 0.011 | 0.942 |
| %12-OH BAs | | 0.229 | 0.015 | -0.049 | 0.610 | 0.183 | 0.202 | -0.052 | 0.720 |
| %Non-12-OH BAs | | -0.229 | 0.015 | 0.049 | 0.610 | -0.183 | 0.202 | 0.052 | 0.720 |
| CA (Conj./Unconj. BAs) | | -0.141 | 0.138 | -0.024 | 0.803 | 0.011 | 0.939 | 0.060 | 0.680 |
| CDCA (Conj./Unconj. BAs) | | -0.143 | 0.133 | -0.057 | 0.547 | 0.124 | 0.393 | -0.013 | 0.929 |
| UDCA (Conj./Unconj. BAs) | | -0.068 | 0.476 | 0.046 | 0.632 | 0.377 | 0.007 | 0.060 | 0.680 |
| LCA (Conj./Unconj. BAs) | | -0.105 | 0.269 | 0.010 | 0.914 | -0.116 | 0.423 | 0.013 | 0.929 |
| DCA (Conj./Unconj. BAs) | | -0.229 | 0.015 | -0.024 | 0.804 | -0.065 | 0.655 | 0.036 | 0.803 |
| GCA/TCA | | -0.078 | 0.416 | 0.066 | 0.491 | 0.086 | 0.553 | -0.095 | 0.511 |
| GCDCA/TCDCA | | -0.067 | 0.486 | 0.055 | 0.565 | -0.112 | 0.438 | -0.073 | 0.613 |
| GUDCA/TUDCA | | -0.048 | 0.617 | 0.110 | 0.247 | -0.155 | 0.284 | 0.050 | 0.732 |
| GLCA/TLCA | | 0.023 | 0.811 | 0.047 | 0.620 | 0.197 | 0.171 | 0.027 | 0.852 |
| GDCA/TDCA | | 0.097 | 0.309 | 0.013 | 0.888 | -0.083 | 0.569 | -0.003 | 0.986 |
| CA/CDCA | | 0.071 | 0.459 | -0.137 | 0.149 | 0.093 | 0.519 | -0.068 | 0.639 |
| LCA/DCA | | -0.172 | 0.070 | 0.019 | 0.842 | 0.070 | 0.629 | -0.026 | 0.857 |
| LCA/UDCA | | 0.192 | 0.043 | 0.048 | 0.613 | 0.305 | 0.031 | 0.005 | 0.974 |
| %CDCAs | | -0.023 | 0.806 | 0.058 | 0.544 | -0.137 | 0.342 | -0.027 | 0.853 |
| %CAs | | 0.121 | 0.204 | -0.176 | 0.064 | -0.022 | 0.879 | 0.032 | 0.826 |
| %DCAs | | 0.186 | 0.049 | 0.027 | 0.774 | 0.251 | 0.079 | -0.032 | 0.826 |
| %UDCAs | | -0.400 | <0.0001 | -0.075 | 0.430 | -0.191 | 0.183 | 0.052 | 0.722 |
| %LCAs | | 0.050 | 0.603 | -0.006 | 0.952 | 0.228 | 0.112 | 0.049 | 0.738 |

**Notes:** NMOSD, neuromyelitis optica spectrum disorder; MS, multiple sclerosis; EDSS, Expanded Disability Status Scale; MRI, magnetic resonance imaging.

**Table S13. Correlation of Bile Acid Biomarkers with EDSS Scores and Spinal Cord MRI T2-Lesions in AQP4-IgG (-) and AQP4-IgG (+)**

| **Indicator** | **AQP4-IgG (-)** | | | | **AQP4-IgG (+)** | | | |  |
| --- | --- | --- | --- | --- | --- | --- | --- | --- | --- |
|  | **r(EDSS)** | ***P*-value** | **r(MRI)** | ***P*-value** | **r(EDSS)** | ***P*-value** | **r(MRI)** | ***P*-value** |  |
| CDCA | | -0.190 | 0.297 | 0.114 | 0.534 | 0.250 | 0.025 | -0.018 | 0.873 |
| GCDCA | | 0.016 | 0.929 | 0.142 | 0.437 | -0.133 | 0.239 | -0.021 | 0.850 |
| TCDCA | | 0.173 | 0.345 | 0.013 | 0.942 | -0.079 | 0.486 | -0.028 | 0.808 |
| CA | | -0.306 | 0.089 | 0.056 | 0.762 | 0.253 | 0.024 | -0.058 | 0.607 |
| GCA | | 0.031 | 0.868 | -0.003 | 0.986 | -0.039 | 0.729 | -0.028 | 0.804 |
| TCA | | 0.195 | 0.284 | -0.124 | 0.501 | -0.040 | 0.724 | -0.100 | 0.378 |
| DCA | | 0.313 | 0.081 | -0.041 | 0.826 | 0.399 | 0.000 | 0.153 | 0.177 |
| GDCA | | 0.255 | 0.158 | -0.138 | 0.450 | 0.054 | 0.631 | 0.138 | 0.223 |
| TDCA | | 0.332 | 0.063 | -0.079 | 0.669 | -0.045 | 0.691 | 0.055 | 0.625 |
| UDCA | | -0.538 | 0.002 | 0.112 | 0.541 | -0.047 | 0.679 | -0.096 | 0.397 |
| GUDCA | | -0.484 | 0.005 | 0.146 | 0.424 | -0.355 | 0.001 | -0.051 | 0.652 |
| TUDCA | | -0.300 | 0.095 | -0.011 | 0.954 | -0.311 | 0.005 | -0.073 | 0.522 |
| LCA | | 0.020 | 0.912 | 0.060 | 0.745 | 0.192 | 0.087 | 0.146 | 0.197 |
| GLCA | | 0.106 | 0.563 | 0.196 | 0.281 | 0.078 | 0.492 | 0.150 | 0.184 |
| TLCA | | 0.171 | 0.350 | 0.023 | 0.902 | 0.086 | 0.448 | 0.101 | 0.375 |
| TBA | | -0.192 | 0.293 | 0.082 | 0.656 | 0.149 | 0.186 | 0.093 | 0.414 |
| %CDCA | | -0.117 | 0.524 | 0.134 | 0.464 | 0.249 | 0.026 | -0.042 | 0.712 |
| %GCDCA | | 0.280 | 0.120 | 0.071 | 0.701 | -0.313 | 0.005 | -0.050 | 0.662 |
| %TCDCA | | 0.305 | 0.090 | -0.153 | 0.402 | -0.169 | 0.134 | -0.074 | 0.515 |
| %CA | | -0.229 | 0.207 | -0.083 | 0.650 | 0.244 | 0.029 | -0.121 | 0.286 |
| %GCA | | 0.343 | 0.054 | -0.221 | 0.225 | -0.128 | 0.258 | -0.067 | 0.556 |
| %TCA | | 0.277 | 0.125 | -0.203 | 0.265 | -0.051 | 0.653 | -0.109 | 0.335 |
| %DCA | | 0.353 | 0.048 | -0.086 | 0.639 | 0.296 | 0.008 | 0.098 | 0.385 |
| %GDCA | | 0.263 | 0.146 | -0.132 | 0.472 | -0.055 | 0.627 | 0.111 | 0.326 |
| %TDCA | | 0.331 | 0.064 | -0.097 | 0.596 | -0.111 | 0.327 | 0.048 | 0.673 |
| %UDCA | | -0.464 | 0.008 | 0.124 | 0.500 | -0.125 | 0.269 | -0.166 | 0.141 |
| %GUDCA | | -0.386 | 0.029 | 0.099 | 0.590 | -0.479 | <0.001 | -0.146 | 0.196 |
| %TUDCA | | -0.175 | 0.338 | -0.095 | 0.604 | -0.429 | <0.001 | -0.157 | 0.165 |
| %LCA | | 0.159 | 0.385 | -0.017 | 0.924 | 0.077 | 0.497 | 0.066 | 0.563 |
| %GLCA | | 0.185 | 0.310 | -0.030 | 0.872 | -0.009 | 0.939 | 0.085 | 0.452 |
| %TLCA | | 0.216 | 0.235 | 0.020 | 0.912 | -0.005 | 0.967 | 0.024 | 0.836 |
| CDCAs | | -0.070 | 0.704 | 0.109 | 0.554 | 0.075 | 0.507 | 0.047 | 0.680 |
| CAs | | -0.141 | 0.442 | -0.065 | 0.723 | 0.173 | 0.124 | -0.022 | 0.848 |
| DCAs | | 0.265 | 0.142 | -0.095 | 0.605 | 0.336 | 0.002 | 0.235 | 0.036 |
| UDCAs | | -0.530 | 0.002 | 0.142 | 0.438 | -0.237 | 0.035 | -0.026 | 0.816 |
| LCAs | | 0.046 | 0.801 | 0.111 | 0.547 | 0.139 | 0.220 | 0.154 | 0.173 |
| 12-OH BAs | | -0.082 | 0.655 | -0.084 | 0.649 | 0.318 | 0.004 | 0.145 | 0.199 |
| Non-12-OH BAs | | -0.188 | 0.302 | 0.094 | 0.610 | 0.015 | 0.895 | 0.018 | 0.874 |
| 12-OH/Non-12-OH BAs | | 0.208 | 0.252 | -0.302 | 0.093 | 0.264 | 0.018 | 0.078 | 0.489 |
| Conjugated CAs | | 0.055 | 0.767 | -0.010 | 0.959 | -0.028 | 0.806 | -0.040 | 0.725 |
| Conjugated CDCAs | | 0.056 | 0.762 | 0.110 | 0.551 | -0.117 | 0.301 | -0.034 | 0.767 |
| Conjugated DCAs | | 0.254 | 0.161 | -0.130 | 0.479 | 0.050 | 0.659 | 0.127 | 0.261 |
| Conjugated UDCAs | | -0.474 | 0.006 | 0.108 | 0.558 | -0.358 | 0.001 | -0.050 | 0.658 |
| Conjugated LCAs | | 0.217 | 0.233 | 0.118 | 0.520 | 0.056 | 0.623 | 0.142 | 0.209 |
| G-Conjugated BAs | | -0.089 | 0.628 | 0.186 | 0.309 | -0.162 | 0.150 | -0.014 | 0.902 |
| T-Conjugated BAs | | 0.192 | 0.291 | -0.013 | 0.942 | -0.068 | 0.548 | -0.023 | 0.837 |
| G/T-Conjugated BAs | | -0.291 | 0.106 | 0.196 | 0.283 | -0.065 | 0.565 | 0.039 | 0.728 |
| Primary BAs | | -0.069 | 0.707 | 0.057 | 0.758 | 0.126 | 0.265 | 0.037 | 0.746 |
| Secondary BAs | | -0.529 | 0.002 | 0.112 | 0.543 | 0.172 | 0.128 | 0.143 | 0.207 |
| Primary/Secondary BAs | | 0.298 | 0.098 | -0.066 | 0.721 | 0.001 | 0.990 | -0.063 | 0.577 |
| Unconjugated BAs | | -0.304 | 0.091 | 0.149 | 0.416 | 0.343 | 0.002 | 0.011 | 0.924 |
| Conjugated BAs | | -0.025 | 0.894 | 0.157 | 0.392 | -0.139 | 0.218 | -0.012 | 0.915 |
| Conjugated/Unconjugated BAs | | 0.210 | 0.249 | -0.004 | 0.982 | -0.355 | 0.001 | -0.007 | 0.952 |
| Neurotoxic BAs | | 0.048 | 0.795 | 0.112 | 0.542 | -0.090 | 0.430 | 0.020 | 0.863 |
| Neuroprotective/Neurotoxic BAs | | -0.476 | 0.006 | 0.094 | 0.609 | -0.175 | 0.121 | -0.103 | 0.365 |
| %Conjugated CAs | | 0.328 | 0.067 | -0.214 | 0.240 | -0.097 | 0.392 | -0.087 | 0.445 |
| %Conjugated CDCAs | | 0.320 | 0.075 | 0.026 | 0.887 | -0.314 | 0.005 | -0.074 | 0.513 |
| %Conjugated UDCAs | | -0.403 | 0.022 | 0.075 | 0.682 | -0.478 | <0.001 | -0.153 | 0.176 |
| %Conjugated LCAs | | 0.202 | 0.268 | 0.025 | 0.892 | -0.077 | 0.496 | 0.045 | 0.691 |
| %Conjugated DCAs | | 0.321 | 0.073 | -0.096 | 0.603 | -0.073 | 0.519 | 0.095 | 0.402 |
| %G-Conjugated BAs | | 0.216 | 0.236 | 0.072 | 0.697 | -0.381 | 0.001 | 0.004 | 0.975 |
| %T-Conjugated BAs | | 0.334 | 0.062 | -0.202 | 0.268 | -0.130 | 0.250 | -0.076 | 0.500 |
| %Unconjugated BAs | | -0.210 | 0.249 | 0.004 | 0.982 | 0.355 | 0.001 | 0.007 | 0.952 |
| %Conjugated BAs | | 0.324 | 0.071 | -0.171 | 0.349 | -0.355 | 0.001 | -0.029 | 0.797 |
| %Primary BAs | | 0.298 | 0.098 | -0.066 | 0.721 | 0.001 | 0.990 | -0.063 | 0.577 |
| %Secondary BAs | | -0.298 | 0.098 | 0.066 | 0.721 | -0.001 | 0.990 | 0.063 | 0.577 |
| %12-OH BAs | | 0.208 | 0.252 | -0.302 | 0.093 | 0.264 | 0.018 | 0.078 | 0.489 |
| %Non-12-OH BAs | | -0.208 | 0.252 | 0.302 | 0.093 | -0.264 | 0.018 | -0.078 | 0.489 |
| CA (Conj./Unconj. BAs) | | 0.312 | 0.082 | -0.056 | 0.762 | -0.282 | 0.011 | 0.034 | 0.764 |
| CDCA (Conj./Unconj. BAs) | | 0.217 | 0.232 | -0.094 | 0.609 | -0.263 | 0.018 | 0.023 | 0.836 |
| UDCA (Conj./Unconj. BAs) | | 0.362 | 0.042 | -0.057 | 0.756 | -0.219 | 0.051 | 0.129 | 0.253 |
| LCA (Conj./Unconj. BAs) | | 0.232 | 0.202 | 0.015 | 0.936 | -0.186 | 0.099 | -0.010 | 0.929 |
| DCA (Conj./Unconj. BAs) | | -0.165 | 0.367 | -0.185 | 0.311 | -0.277 | 0.013 | -0.038 | 0.736 |
| GCA/TCA | | -0.126 | 0.494 | 0.149 | 0.417 | -0.042 | 0.712 | 0.106 | 0.350 |
| GCDCA/TCDCA | | -0.233 | 0.199 | 0.163 | 0.373 | 0.002 | 0.989 | 0.079 | 0.487 |
| GUDCA/TUDCA | | -0.192 | 0.293 | 0.283 | 0.117 | 0.011 | 0.923 | 0.087 | 0.445 |
| GLCA/TLCA | | -0.073 | 0.691 | 0.099 | 0.589 | -0.021 | 0.856 | 0.035 | 0.757 |
| GDCA/TDCA | | 0.106 | 0.565 | -0.109 | 0.553 | 0.074 | 0.513 | 0.083 | 0.464 |
| CA/CDCA | | -0.238 | 0.189 | -0.170 | 0.351 | 0.139 | 0.219 | -0.056 | 0.621 |
| LCA/DCA | | -0.339 | 0.058 | 0.052 | 0.776 | -0.208 | 0.064 | -0.037 | 0.745 |
| LCA/UDCA | | 0.467 | 0.007 | -0.031 | 0.868 | 0.120 | 0.288 | 0.133 | 0.239 |
| %CDCAs | | 0.219 | 0.229 | 0.088 | 0.633 | -0.079 | 0.487 | 0.000 | >0.999 |
| %CAs | | 0.129 | 0.482 | -0.265 | 0.142 | 0.105 | 0.354 | -0.104 | 0.358 |
| %DCAs | | 0.299 | 0.096 | -0.085 | 0.642 | 0.215 | 0.055 | 0.157 | 0.164 |
| %UDCAs | | -0.399 | 0.024 | 0.174 | 0.341 | -0.377 | 0.001 | -0.149 | 0.188 |
| %LCAs | | 0.184 | 0.313 | 0.032 | 0.863 | -0.012 | 0.919 | 0.052 | 0.648 |

**Notes:** NMOSD, neuromyelitis optica spectrum disorder; EDSS, Expanded Disability Status Scale; MRI, magnetic resonance imaging; AQP4-IgG, anti–aquaporin-4 immunoglobulin G.

**Supplementary Figure**


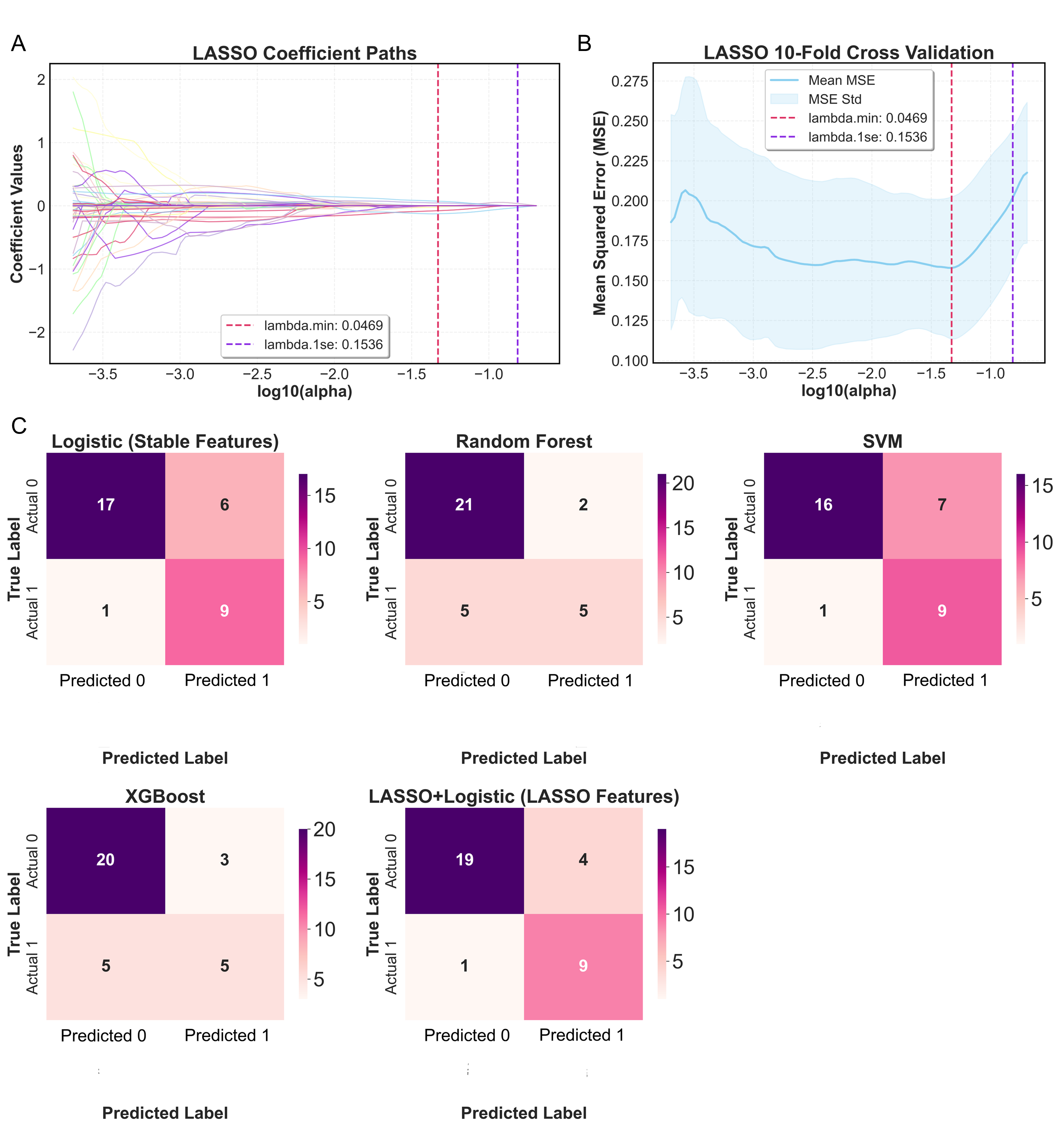


**Figure S1. Model Development and Evaluation.**

**(A)** Coefficient trajectories across different regularization parameters (logλ). Vertical dashed lines indicate lambda.min (red) and lambda.1se (blue) values selected through 10-fold cross-validation. **(B)** Mean squared error (MSE) ± standard error across regularization parameters. Lambda.min (red dashed line) represents the value giving minimum cross-validation error, while lambda.1se (blue dashed line) represents the most regularized model within one standard error of the minimum. **(C)** Confusion matrices for all machine learning modelsConfusion matrices display classification performance at optimal decision thresholds for SVM, LASSO+Logistic, Logistic Regression with stable features, Random Forest, and XGBoost models on the independent test set.
